# Catalytic asymmetric oxo-Diels–Alder reactions with chiral atropisomeric biphenyl diols

**DOI:** 10.3762/bjoc.15.92

**Published:** 2019-04-18

**Authors:** Chi-Tung Yeung, Wesley Ting Kwok Chan, Wai-Sum Lo, Ga-Lai Law, Wing-Tak Wong

**Affiliations:** 1 The Hong Kong Polytechnic University Shenzhen Research Institute, Shenzhen, PR China; 2 State Key Laboratory of Chemical Biology and Drug Discovery, Department of Applied Biology and Chemical Technology, The Hong Kong Polytechnic University, Hung Hom, Kowloon, Hong Kong

**Keywords:** asymmetric organocatalysis, axial chirality, biaryls, hydrogen bond, oxo-Diels–Alder reaction

## Abstract

New chiral atropisomeric biphenyl diols **3**, **4** and **6** containing additional peripheral chiral centers with different steric bulkiness and/or electronic properties were synthesized. The X-ray crystal structure of **3** shows the formation of a supramolecular structure whereas that of **6**, containing additional CF_3_ substituents, shows the formation of a monomeric structure. Diols **1**–**6** were found to be active organocatalysts in oxo-Diels–Alder reactions in which **2** recorded a 72% ee with trimethylacetaldehyde as a substrate.

## Introduction

The Diels–Alder (DA) reaction is a useful and easy-to-perform method for the synthesis of six-membered rings through the direct formation of C–C bonds between a diene and a dienophile (a substituted alkene); it is called a hetero-Diels–Alder (HDA) reaction when one or more heteroatoms (most often oxygen or nitrogen) are present among the reactants, such as the use of carbonyl compounds or imines as dienophiles [1–5]. An asymmetric HDA reaction is capable of introducing up to four stereogenic centers in a one-step [4 + 2] cycloaddition or cyclization reaction [6–8] and it has become hugely popular in preparing vital intermediates for the syntheses of key structural subunits of natural products with biological activities (e.g., carbohydrates, antibiotics, toxins etc.) [9–10]. Alternate synthetic pathways include ring formations of open-chained precursors [11–12], reactions of dicarbonyl compounds with ketene diethylacetal followed by hydrolysis [13] or total syntheses [14]; however, none of these alternatives could rival the combination of ease and cost-effectiveness of HDA reactions.

Oxo-Diels–Alder (oxo-DA) reactions between electron-poor aldehydes and electron-rich dienes such as Danishefsky’s dienes or Brassard’s dienes are efficient ways to construct oxygen-containing six-membered heterocycles via [4 + 2] cyclizations, and have been dominated by metal-based chiral Lewis acid catalysts for over three decades [15–26]. Comparatively, interest in the utilization of metal-free organocatalytic oxo-DA reactions began to grow only after Rawal’s group reported a ground-breaking contribution in using a diol molecule, TADDOL, as a hydrogen bonding organocatalyst for the reaction between 1-dimethylamino-3-*tert*-butyldimethylsilyloxy-1,3-butadiene (Rawal’s diene) and aldehydes with excellent enantioselectivities (aromatic aldehyde: up to 86–98% ee) in 2003 [27]. Activation via a single-point hydrogen bond between one of the hydroxy groups of the TADDOL and the carbonyl oxygen of the aldehyde was proposed to be a crucial factor for the success of this organocatalyst in the reaction. Two years later, they reported another efficient diol-based hydrogen bonding organocatalyst, BAMOL, for catalyzing the same oxo-DA reactions with a library of aldehydes (aromatic: 97–99% ee; aliphatic: 84–98% ee) [28]. Thereafter, many different kinds of hydrogen bonding-based organocatalysts have been developed for oxo-DA reactions [29–35]. One kind of organocatalyst in particular, which is based on an oxazoline template with hydroxy and NH units for hydrogen bonding activation, % ee and yields in oxo-DA reactions were found to be enhanced with increasing NH acidity, leading to stronger hydrogen bonds [32]. So, there is much room for further investigation and improvement with other hydrogen bonding organocatalysts.

**Scheme 1:**
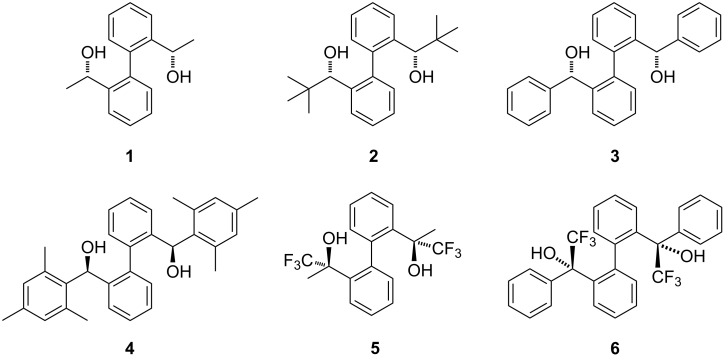
Chiral biphenyl diol organocatalysts **1–6**.

An earlier work by Goldfuss and his co-worker on an atropisomeric biphenyl compound showed that atropisomerism can be induced and stabilized with hydrogen bonding from fenchyl alcoholic units [36]. Regarding to atropisomeric properties of biphenyl compounds, our group have previously reported the formation of supramolecular helices or dimers through intermolecular hydrogen bonding of two axially chiral biphenyl hybrid diols (**1** and **2** in Scheme 1) which contain point chirality at the side arms and axial chirality at the biphenyl backbone [37]. We envisage the structural similarity and the ability of our scaffold to form strong hydrogen bonds could perform the same catalytic role in oxo-DA reactions as reported in the literature. Inspired by Rawal’s work on TADDOL and BAMOL organocatalysts, we, in this work, have adopted a similar reaction of Rawal’s diene with benzaldehyde as a starting point of our study and other biphenyl hybrid diols with different steric bulky substituents were incorporated (**3** and **4**). Since it is known that the catalyst acidity has significant influence on hydrogen-bond-catalyzed reactions [32], we also incorporated a CF_3_ group in our molecular scaffold to investigate how it would affect the reactivity and selectivity (**5** and **6**). Recently, we found that compound **5**, which can form a pair of atropisomer (*P*)-(*R*,*R*)-**5** and (*M*)-(*R*,*R*)-**5**, gave different results in *N*-nitroso aldol reactions compared to **1** [38].

## Results and Discussion

### Synthetic procedures

**Scheme 2:**
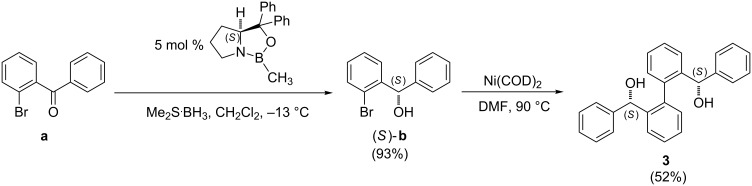
Synthesis of **3**.

**Figure 1:**
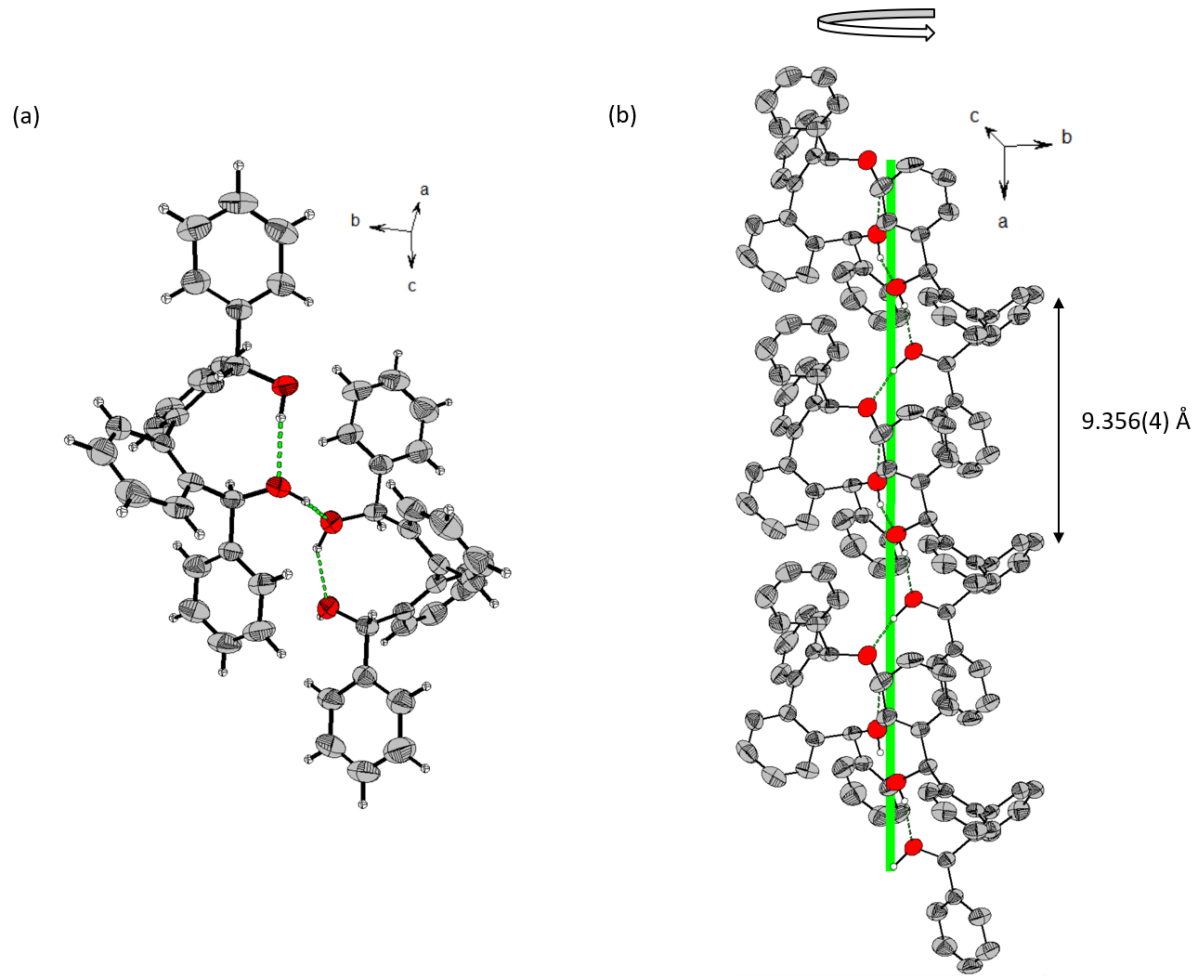
(a) Single crystal X-ray structure of **3**: showing intra- and intermolecular hydrogen bonds (green dashed line). Torsion angles of biphenyl rings is 76.25(35)–78.27(35)°; axial configuration is *M*. (b) Crystal packing of **3** shows one strand of supramolecular left-handed helixes (hydrogens that are not involved in interactions are omitted for clarity).

Synthetic steps of catalyst **3** were similar to that for **1** and **2** [37]. Asymmetric reduction of the carbonyl group of 2’-bromobenzophenone (**a**) with borane dimethyl sulfide in the presence of (*S*)-(−)-2-methyl-CBS-oxazaborolidine catalyst gave (*S*)-(2-bromophenyl)(phenyl)methanol (*S*)-**b** in 93% yield and 94% ee (Scheme 2 and Figure S1 in Supporting Information File 1). Homo-coupling of (*S*)-**b** with Ni(COD)_2_ was achieved with very high diastereoselectivity and only a single atropisomer was formed. The X-ray crystal structure showed that it is (*M*)-(*S*,*S*)-**3** (Figure 1). This high atropstereoselectivity is believed to be due to the energetically unfavorable formation of the other atropisomer (*P*)-(*S*,*S*)-**3** as a higher steric repulsion is generated during the close approach of two bulky phenyl peripheral substituents. The X-ray crystal structure also reveals that an alternative formation of intra- and intermolecular hydrogen bonds [intra- D(OH···O): 1.951(3) Å; inter- D(OH···O): 1.822(3) Å] led to an enantiomerically pure infinite helical supramolecular structure. In contrast, only a dimeric structure was observed for the corresponding racemic mixture [39]. In our case, atropisomerization from (*M*)-(*S*,*S*) to (*P*)-(*S*,*S*) was not observed in solution.

**Scheme 3:**
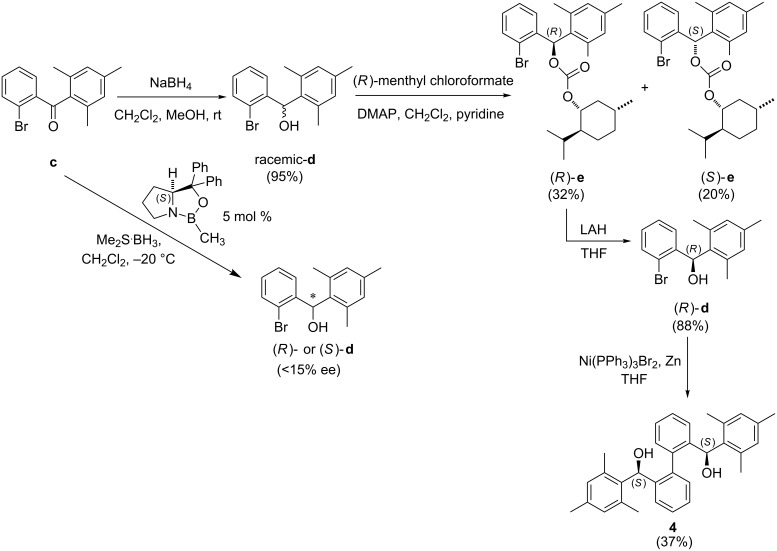
Synthesis of **4**.

For catalyst **4**, different from the synthesis of catalyst **3**, asymmetric reduction of the corresponding ketone, (2-bromophenyl)(mesityl)methanone **c**, using (*S*)-(–)-2-methyl-CBS-oxazaborolidine as the catalyst resulted in a very low enantioselectivity (<15% ee) of the product, (2-bromophenyl)(mesityl)methanol (**d**, Scheme 3). For this reason, another strategy of obtaining high % ee of **d** was employed. Chiral resolution of racemic-**d** with (*R*)-menthyl chloroformate gave a high % ee of this key intermediate **d**. After column chromatographic purification of the crude reaction mixture, the corresponding diastereomers (*R*)-**e** or (*S*)-**e** can be partly separated. Their diastereoselectivities can be further enhanced by slow evaporation of the corresponding diastereomers from acetonitrile and the absolute configuration of one diastereomer was confirmed with X-ray crystal structure (Supporting Information File 1, Figure S2). After removing the chiral-resolving menthyl substituent with lithium aluminium hydride (LAH), the enantioselectivity of (*R*)-**d** was higher than 99% ee when checked with HPLC (Supporting Information File 1, Figure S3). Catalyst **4** was then obtained by homo-coupling of (*R*)-**d** with Ni(PPh_3_)_3_Br_2_/Zn with 37% yield. The low yield of catalyst **4** may be due to steric bulkiness of the mesityl group that hampers close approach of **d** during coupling.

**Scheme 4:**
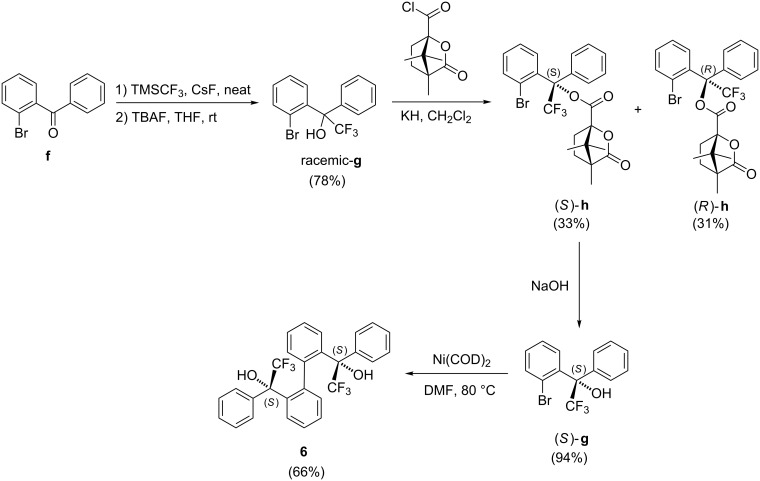
Synthesis of **6**.

**Figure 2:**
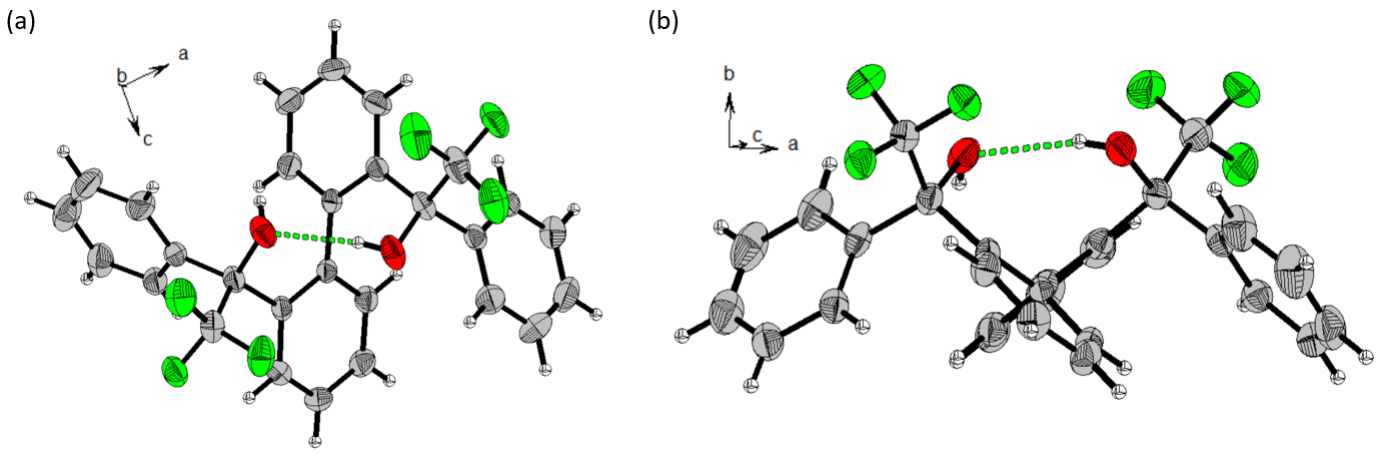
X-ray crystal structure of (*P*)-(*S*,*S*)-**6** at two different orientations to show (a) *P* atropselectivities; (b) different relative spatial arrangement of the CF_3_- and phenyl-substituents. Torsional angle of biphenyl rings is 104.55(22).

For the synthesis of catalyst **6**, the CF_3_ substituent was introduced to **f** by trifluoromethylation (Scheme 4). A racemic mixture of **g** was obtained after the TMS group was removed with TBAF. This racemate then underwent chiral resolution by reacting with (1*S*)-(−)-camphanic chloride to give a diastereomeric mixture of (*R*)-**h** or (*S*)-**h**. After separating these diastereomers by column chromatography, the absolute configuration of (*S*)-**h** was checked with its X-ray crystal structure (Supporting Information File 1, Figure S4). The optically pure (*S*)-**g** was then obtained by removing the chiral camphanic substituent (Supporting Information File 1, Figure S5) and (*S*)-**g** was then utilized for homo-coupling with Ni(COD)_2_ to give catalyst **6**. X-ray crystal structure of **6** showed that it is an (*P*)-(*S*,*S*) atropisomer (Figure 2). From the structure, no extensive intermolecular OH···O hydrogen bonds can be found for the formation of the supramolecular structure. Instead, intramolecular hydrogen bonds [D(O–H···O): 2.072(31) Å; 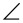
(O–H∙∙∙O): 161.39(31)°] were found to keep the conformation of the biphenyl ring intact. Relative spatial arrangement of the larger phenyl substituent is located at the side where it is relatively far away from the intramolecular hydrogen bond unit.

### Catalytic studies

With the organocatalyst **1** in hand, we firstly examined the oxo-DA reaction of benzaldehyde and Rawal’s diene in standard conditions. The reaction was found to be very sluggish when performed at −40 °C. However, at −20 °C, better yields were obtained. The reactions with organocatalysts **1–4** showed moderate yields (41–64%) of the catalytic product (2,3-dihydro-2-phenyl-4*H*-pyran-4-one). Unfortunately, benzaldehyde was found to be a challenging substrate and low enantioselectivities (2–8% ee) (Table 1, entries 1–4) were obtained. The enantioselectivities can be improved slightly (32% ee and 11% ee) when (*P*)-(*R*,*R*)-**5** and (*M*)-(*R*,*R*)-**5** were employed as catalysts, although the yields decreased sharply (Table 1, entries 5 and 6). It should be noted that the absolution configuration of the catalytic product seems to be determined by the axial chirality (*P* or *M*) of **5** rather than its point chirality at the side arms (both have the same *R*-chirality) [40]. The axial chirality of other catalysts have been reported previously to have a significant effect on controlling catalytic enantioselectivites [41–42]. For organocatalyst **6**, it gave the highest enantioselectivity (59% ee) but a low chemical yield.

**Table 1:** Catalytic asymmetric oxo-DA reactions with stereolabile chiral biphenyl diols **1–6**.^a^

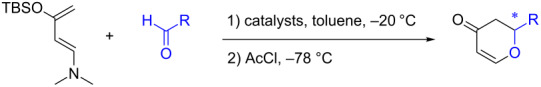				

Entry	Catalyst	Aldehyde	Yield (%)^b^	ee (%) (configuration)^c^

1	**1**	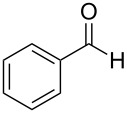	46	7 (*S*)
2	**2**		41	2 (*S*)
3	**3**		52	3 (*S*)
4	**4**		64	8 (*R*)
5	(*P*)-(*R*,*R*)-**5**		15	32 (*S*)
6	(*M*)-(*R*,*R*)-**5**		16	11 (*R*)
7	**6**		15	59 (*R*)

8	**1**	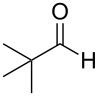	50	12^e^
9	**2**		66	72^e^
10^d^	**2**		26	74^e^
11	**3**		28	16^e^
12	**4**		16	6^e^
13	(*P*)-(*R*,*R*)-**5**		14	30^e^
14	(*M*)-(*R*,*R*)-**5**		15	10^e^
15	**6**		16	10^e^

16	**2**	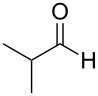	42	56 (*R*)
17	**2**	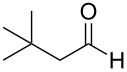	32	52^f^
18	**2**	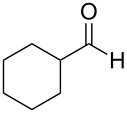	33	43(*R*)
19	**2**	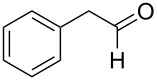	30	36^f^
20	**2**	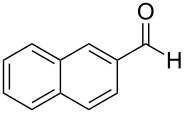	25	2^f^

^a^Reactions were run with 0.1 mmol of catalyst, 1.0 mmol aldehyde and 0.5 mmol diene in 0.5 mL dried toluene under nitrogen at −20 °C for 1 day. Then the reactions were worked up with 1 mmol of AcCl at −78 °C. Products were isolated by column chromatography with silica gel. ^b^Isolated yields. ^c^Determined with HPLC with chiral columns, and the absolute configuration assigned by comparison with the order of elution of known compounds [28–29,43]. ^d^Reaction was performed at −40 °C for 3 days. ^e^Absolute configuration undetermined. OD-H column, Hex:IPA = 98:2, 1 mL/min, (10.7 min and 11.6 min) latter peak is a major peak for entries 8–12, 14 and 15. Previous peak is a major peak for entry 13. ^f^Absolute configuration undetermined.

When the bulkier trimethylacetaldehyde was used as substrate, the enantioselectivities were improved when **1–3** were used as catalysts (Table 1, entries 8–11). With **1**, the yield and enantioselectivity of the catalytic product (2,3-dihydro-2-*tert*-butyl-4*H*-pyran-4-one) were 50% and 12% ee (Table 1, entry 8). The reactivity and enantioselectivity were significantly increased to 66% yield and 72% ee when **2** was employed as the catalyst (Table 1, entry 9). The enantioselectivity can be improved slightly to 74% when the temperature was decreased to −40 °C (Table 1, entry 10). For **3** and **4**, with aromatic substituents, the yields and enantioselectivities of the catalytic product decreased dramatically from **2** (Table 1, entries 11 and 12). For **5**, similar to the case of using benzaldehyde as substrate, (*P*)-(*R*,*R*)-**5** resulted in a higher enantioselectivity than (*M*)-(*R*,*R*)-**5** and their products have opposite absolute configurations (Table 1, entries 13 and 14). For organocatalyst **6**, the enantioselectivity was not as good as when using benzaldehyde as substrate (Table 1, entry 15).

In order to explore the scope of the present oxo-DA reaction, we next examined the reactions of other aldehydes with **2** as the catalyst. With less bulky aliphatic aldehydes, which only have hydrogens at the alpha positions such as isobutyraldehyde, 3,3-dimethylbutyraldehyde, cyclohexylaldehyde and 2-phenylacetaldehyde, decrease in the yields and enantioselectivities were observed (Table 1, entries 16–19). With another aromatic aldehyde, 2-naphthaldehyde, a racemic product was obtained.

## Conclusion

Three new chiral atropisomeric biphenyl diols **3**, **4** and **6** with axial chiralities controlled by their corresponding additional asymmetric carbon centers were synthesized; despite having the same biphenyl scaffold, their highly enantioselective intermediates **b**, **d** and **g** were obtained with different strategies: asymmetric reduction with oxazaborate catalyst for **3**, chiral resolution with (*R*)-menthyl chloroformate for **4** and chiral resolution with (1*S*)-camphanic chloride for **6**. Crystal structures revealed that the presence and absence of additional CF_3_ substituents in **3** and **6** led to very different structures, as **3** forms helical supramolecular structure with continuous and alternative inter- and intramolecular hydrogen bonds, whereas **6** forms a monomer without intermolecular hydrogen bonds for supramolecular formation. Together with compounds **1**, **2** and **5**, all were found to be active organocatalysts in oxo-DA reactions, with **2** resulting in the highest reactivity and enantioselectivity with trimethylacetaldehyde as a substrate. Opposite absolute configurations of the catalytic products of benzaldehyde from atropisomers of **5** showed that axial chirality contributes significantly to high enantioselectivities. Further works on organocatalyst optimization with different substituents are ongoing in our group and further experiments are underway to develop the use of these diols for other catalytic reactions.

## Supporting Information

File 1 Experimental data.
