# Supplementary material for: Catalytic asymmetric oxo-Diels–Alder reactions with chiral atropisomeric biphenyl diols
Source: Beilstein J Org Chem. 2019 Apr 18;15:955–62. doi: 10.3762/bjoc.15.92 (PMC6541354; doi:10.3762/bjoc.15.92)
Supplement: File 1 — Experimental data. [file Beilstein_J_Org_Chem-15-955-s001.pdf]

BEILSTEIN JOURNAL OF ORGANIC CHEMISTRY

Supporting Information

for

Catalytic asymmetric oxo-Diels–Alder reactions with chiral atropisomeric biphenyl diols

Chi-Tung Yeung, Wesley Ting Kwok Chan, Wai-Sum Lo, Ga-Lai Law and Wing-Tak Wong

Beilstein J. Org. Chem. **2019,** *15,* 955–962. doi:10.3762/bjoc.15.92

Experimental data

License and Terms: This is a supporting information file under the terms of the Creative Commons Attribution License (http://creativecommons.org/licenses/by/4.0). Please note that the reuse, redistribution and reproduction in particular requires that the authors and source are credited.
The license is subject to the *Beilstein Journal of Organic Chemistry* terms and conditions: (https://www.beilstein-journals.org/bjoc)

Experimental

General

(1*S*,1'*S*)-1,1'-(biphenyl-2,2'-diyl)diethanol (**1**) and (1*S*,1'*S*)-1,1'-(biphenyl-2,2'-diyl) bis(2,2-dimethylpropan-1-ol) (**2**) were prepared accordingly to previous report [1]. Solvents were dried using an appropriate drying agent when required (benzene and hexane over sodium, tetrahydrofuran and diethyl ether over sodium/benzophenone, dichloromethane over calcium hydride). Anhydrous dimethylformamide was available commercially. Other chemicals were purchased commercially and used as received. Unless otherwise stated, all manipulations were carried out under nitrogen using Schlenk line technique. NMR spectra were recorded on a Bruker Ultrashield Avance Pro 400 MHz instrument. Chemical shifts of ^{1}H and ^{13}C were referenced internally to tetramethylsilane or solvent residual peaks in deuterated solvents in parts per million (ppm). Chemical shifts of ^{19}F were referenced externally with trifluoroacetic acid (−76.55 pm). The absolute configuration was determined with X-ray crystallography.

The crystal data reported were either collected on a Bruker Smart 1000 diffractometer system equipped with an APEX II CCD detector [**3** and (*R*)-**e**] or a Bruker D8-Venture system [**6** and (*S*)-**h**]. The four structures were collected with a Mo Kα radiation. All data were collected at room temperature. Multi-scan absorption correction was applied by SADABS program [2], and the SAINT program, Bruker-AXS 2014 APEX3 software suite (Madison, Wisconsin, USA), utilized for the integration of the diffraction. All structures were solved by direct method and were refined by a full-matrix least-squares treatment on F^2 using the SHELXLE programme system [3]. The crystallographic data for the

structural analyses have been deposited with the Cambridge Crystallographic Data Centre, CCDC No. and the data can be obtained free of charge via www.ccdc.cam.ac.uk/data_request/cif. CCDC No. 1866715 for **3**; 1866734 for **6**; 1866717 for (*R*)-**e** and 1866716 for (*S*)-**h**.

Syntheses

(*S*)-(2-Bromophenyl)(phenyl)methanol, ((*S*)-b). 2-Bromobenzophenone, **a**, (1.10 g, 4.20 mmol) in CH_2Cl_2 (0.9 mL) was added dropwisely to a solution of oxazaborole-borane catalyst (0.06 g, 0.21 mmol) and borane-dimethyl sulfide (0.40 mL, 4.15 mmol) in dichloromethane (0.30 mL) at –13 ^{o}C. After stirring the reaction mixture at this temperature for 16 h, the mixture was precooled to –20 ^{o}C and then quenched carefully with dropwise addition of methanol until no effervescence was observed. After evaporating all the solvents, the crude product was purified with column chromatography to yield colourless gel of (*S*)-**b** (1.15 g, 4.39 mmol, 93% yield). ^{1}H NMR (400 MHz, $CDCl_3$, δ): 2.36 (d, *J* = 4.0 Hz, 1H), 6.20 (d, *J* = 4.0 Hz, 1H), 7.14 (t, *J* = 4.0 Hz, 1H), 7.26 (d, J = 4.0 Hz, 1H), 7.31–7.34 (m, 3H), 7.39 (d, *J* = 8 Hz, 2H), 7.53 (d, *J* = 8H, 1H), 7.57 (d, *J* = 8H, 1H). ^{13}C NMR (100 MHz, $CDCl_3$, δ): 74.80, 122.82, 127.01, 127.71, 127.76, 128.46, 128.51, 129.10, 132.85, 142.17, 142.54.

(1*S*,1'*S*)-Biphenyl-2,2'-diylbis(phenylmethanol), (3). (*S*)-(2-bromophenyl)(phenyl)methanol, (*S*)-**b**, (0.50 g, 1.90 mmol) was added to a suspension of bis(1,5-cyclooctadiene)nickel(0) (0.26 g, 0.95 mmol) in 2.4 mL anhydrous dimethylformamide under nitrogen. The mixture was heated at 90 ^{o}C for 16 h. After cooling to room temperature,

the reaction was quenched by addition of 5% aqueous hydrochloric acid and then extracted with diethyl ether (20 mL × 3). The extract was dried with anhydrous magnesium sulfate, filtered, concentrated. The residue was purified with column chromatography (petroleum ether/ ethyl acetate, 10:1) to yield **3** (0.18 g, 0.49 mmol, 52% yield). Crystals were formed by slow evaporation of the product from diethyl ether solution. ^{1}H NMR (400 MHz, $CDCl_3$, δ): 3.41 (s, 2H), 5.69 (d, *J* = 2 Hz, 2H), 7.23–7.33 (m, 18H). ^{13}C NMR (100 MHz, $CDCl_3$, δ): 71.80, 126.05, 127.03, 127.46, 128.09, 128.15, 128.39, 129.61, 140.00, 142.46, 143.22.

Racemic (2-bromophenyl)(mesityl)methanol, (racemic-d). Sodium borohydride (0.087 g, 2.29 mmol) was added to a solution of (2-bromophenyl)(mesityl)methanone, (**c**, 0.20 g, 0.66 mmol) in dichloromethane (6 mL) and methanol (2 mL). After stirring the reaction mixture at room temperature for 3 h, 20 mL of water was added. The product, racemic-**d**, was then extracted with diethyl ether and then used for next step without further purification (0.64 g, 2.18 mmol, 95% yield). ^{1}H NMR (400 MHz, $CDCl_3$, δ): 2.24 (s, 6H), 2.28 (s, 3H), 2.46 (d, *J* = 4 Hz, 1H), 6.30 (d, *J* = 4 Hz, 1H), 6.85 (s, 2H), 7.13 (t, *J* = 8 Hz, 1H), 7.22–7.26 (m, 1H), 7.37 (d, *J* = 8 Hz, 1H), 7.55 (d, *J* = 8 Hz, 1H). ^{13}C NMR (100 MHz, $CDCl_3$, δ): 20.82, 21.19, 72.38, 123.34, 127.12, 128.91, 129.38, 130.15, 133.07, 133.94, 137.10, 137.30, 141.35.

(*R*)-(2-Bromophenyl)(mesityl)methyl (1*R*,2*S*,5*R*)-2-isopropyl-5-methylcyclohexyl carbonate, ((*R*)-e) and (*S*)-(2-bromophenyl)(mesityl)methyl (1*R*,2*S*,5*R*)-2-isopropyl-5-methylcyclohexyl carbonate, ((*S*)-e). (*R*)-Menthyl chloroformate (4.94 g, 22.59 mmol) was added to a solution

of (2-bromophenyl)(mesityl)methanol, (racemic-**d**, 2.30 g, 7.53 mmol), pyridine (1.83 mL, 22.59 mmol) and 4-(dimethylamino)pyridine (0.92 g, 0.753 mmol) in anhydrous dichloromethane (150.0 mL) at 0 ^{o}C. After stirring the reaction mixture at room temperature for 16 h, the mixture was diluted with ethyl acetate (100 mL), and then washed with HCl (1 M, 50 mL x 3) and saturated aqueous potassium carbonate (50 mL). The organic layer was then dried with magnesium sulfate, filtered and concentrated. The crude residue was then purified with column chromatography (petroleum ether/benzene, 25:1) to give (*S*)-**e**, [(*S*)-(2-bromophenyl)(mesityl)methyl (1*R*,2*S*,5*R*)-2-isopropyl-5-methylcyclohexyl carbonate] (0.729 g, 1.49 mmol, 20% yield) and (*R*)-**e**, [(*R*)-(2-bromophenyl)(mesityl)methyl(1*R*,2*S*,5*R*)-2-isopropyl-5-methyl cyclohexyl carbonate] (1.16 g, 2.38 mmol, 32% yield). Purity of the two isomers was further enhanced with recrystallization by slow evaporation of the corresponding isomer from acetonitrile. For (*S*)-**e**: ^{1}H NMR (400 MHz, $CDCl_3$, δ): 0.80 (d, *J* = 8 Hz, 3H), 0.86–0.90 (m, 7H), 0.99–1.05 (m, 2H), 1.40-1.43 (m, 2H), 1.64–1.67 (m, 2H), 1.96–2.04 (m, 2H), 2.28 (s, 9H), 4.56 (td, *J* = 11.2, 4.4 Hz, 1H), 6.89 (s, 2H), 6.97–7.00 (m, 1H), 7.15–7.17 (m, 3H), 7.62–7.64 (m, 1H). ^{13}C NMR (100 MHz, $CDCl_3$, δ): 16.08, 20.73, 20.89, 21.00, 21.89, 23.11, 25.87, 31.41, 34.10, 40.77, 47.05, 78.46, 125.51, 127.12, 129.96, 130.11, 130.24, 130.40, 133.56, 136.19, 137.12, 137.77, 154.27. For (*R*)-**e**: ^{1}H NMR (400 MHz, $CDCl_3$, δ): 0.68 (d, *J* = 8 Hz, 3H), 0.85 (m, 4H), 0.92 (d, *J* = 4 Hz, 3H), 0.96–1.08 (m, 2H), 1.38–1.51 (m, 2H), 1.64–1.67 (m , 2H), 1.91–1.93 (m, 1H), 2.15–2.18 (m, 1H), 2.29 (s, 9H), 4.45 (td, *J* = 11.2, 4.4 Hz, 1H), 6.89 (s, 2H),

6.98–7.00 (m, 1H), 7.15–7.18 (m, 3H), 7.62–7.64 (m, 1H). ^{13}C NMR (100 MHz, $CDCl_3$, δ): 16.28, 20.62, 20.89, 20.94, 21.95, 23.43, 26.13, 31.38, 34.15, 40.58, 46.81, 78.75, 125.44, 127.12, 129.96, 130.09, 130.21, 130.37, 133.54, 136.23, 137.11, 137.77, 154.23. Crystals of the (*R*)-**e** were formed by slow evaporation of the product from diethyl ether solution. Crystals are good enough for X-ray crystal crystallography.

(*R*)-2-Bromophenyl(mesityl)methanol, ((*R*)-d). Lithium aluminum hydride (2.4M in THF) (1.75 mL, 4.2 mmol) was added to a solution of (*R*)-(2-bromophenyl)(mesityl)methyl(1*R*,2*S*,5*R*)-2-isopropyl-5-methyl cyclohexylcarbonate ((*R*)-**e**, 0.20 g, 0.4 mmol) in anhydrous THF (19 mL) at 0 ^{o}C. After stirring the reaction mixture at room temperature for 16 h, 10 mL of 10% HCl was added carefully. The organic layer was extracted with diethyl ether (10 mL × 3), then dried with magnesium sulfate, filtered and concentrated. The residue was purified with column chromatography (hexane/isopropanol, 9:1) to yield enantiomerically pure (*R*)-(2-bromophenyl)(mesityl)methanol ((*R*)-**d**, 0.11g, 0.35 mmol, 88% yield). ^{1}H NMR (400 MHz, $CDCl_3$, δ): 2.25 (s, 6H), 2.28 (s, 3H), 2.47 (d, *J* = 4 Hz, 1H), 6.31 (d, *J* = 4 Hz, 1H), 6.86 (s, 2H), 7.13 (t, *J* = 8 Hz, 1H), 7.23–7.27 (m, 1H), 7.38 (d, *J* = 8 Hz, 1H), 7.56 (d, *J* = 8 Hz, 1H). ^{13}C NMR (100 MHz, $CDCl_3$, δ): 20.82, 21.19, 72.41, 123.35, 127.13, 128.91, 129.39, 130.17, 133.09, 133.97, 137.11, 137.31, 141.40.

(1*S*,1'*S*)-Biphenyl-2,2'-diylbis(mesitylmethanol), (4). Zinc dust (0.075 g, 1.15 mmol) was added to a solution of dibromobis(triphenylphosphine)nickel(II) (0.085 g, 0.12 mmol) and tetramethylammonium iodide (0.29 g, 1.15 mmol) in anhydrous THF (1.4 mL) at room temperature. After stirring the reaction mixture at room

temperature for 1 h, (*R*)-(2-bromophenyl)(mesityl)methanol ((*R*)-**d**, 0.18 g, 0.57 mmol) in THF (1.1 mL) was added and then the reaction mixture was heated at 60 ^{o}C for 16 h. After cooling to room temperature, the reaction was quenched by addition of 5% aqueous hydrochloric acid and then extracted with diethyl ether. The extract was dried with anhydrous magnesium sulfate, filtered, concentrated. The residue was purified with column chromatography (petroleum ether/ ethyl acetate, 15:1) to give **4** (0.048 g, 0.11 mmol, 37% yield). ^{1}H NMR (400 MHz, CD_3OD, δ): 2.12 (s, 6H), 2.23 (s, 3H), 6.30 (s, 1H), 6.75 (s, 2H), 6.86 (d, *J* = 8 Hz, 1H), 7.11–7.15 (m, 1H), 7.20 (d, J = 4 Hz, 2H). ^{13}C NMR (100 MHz, CD_3OD, δ): 20.91, 21.51, 71.19, 127.88, 128.01, 128.78, 130.49, 130.77, 136.68, 137.64, 138.32, 140.68, 142.63.

1-(2-Bromophenyl)-2,2,2-trifluoro-1-phenylethanol, (racemic-g). Cesium fluoride (0.03 g, 0.20 mmol) was added to a solution of 2-bromobenzophenone (**f**, 2.3 g, 8.81 mmol) and (trifluoromethyl)trimethylsilane (2.0 mL, 13.55 mmol) at 0 ^{o}C with stirring. Effervescence was observed. After no bubble appeared, the reaction mixture was stirred at room temperature for 16 h. After rotary evaporating the excess reagent, tetrahydrofuran (10 mL) and then tetrabutylammonium fluoride (70%, 16 mL) were added subsequently to the residue. After stirring the reaction mixture at room temperature for 1 h, the mixture was diluted with water (50 mL), and the product was extracted with diethyl ether (30 mL × 3). The extract was dried with anhydrous magnesium sulfate and purified with column chromatography (petroleum ether/ ethyl acetate, 40:1) to yield 1-(2-bromophenyl)-2,2,2-trifluoro-1-phenylethanol (racemic-**g**, 2.3 g,

6.87 mmol, 78% yield). ^{1}H NMR (400 MHz, $CDCl_3$, δ): 3.95 (s, 1H), 7.25 (t, *J* = 8 Hz, 1H), 7.32–7.38 (m, 5H), 7.44 (t, *J* = 4 Hz, 1H), 7.60 (d, *J* = 4 Hz, 1H), 7.83 (d, *J* = 4 Hz, 1H). ^{13}C NMR (100 MHz, $CDCl_3$, δ): 81.11 (q, *J* = 29 Hz), 122.84, 124.55 (q, *J* = 285 Hz), 127.17, 127.90, 127.96, 128.67, 129.27(q, *J* = 5 Hz), 130.22, 135.94, 137.47, 137.98. ^{19}F NMR (376 MHz, $CDCl_3$, δ): –72.46 (s).

(1*S*,4*R*)-((*S*)-1-(2-Bromophenyl)-2,2,2-trifluoro-1-phenylethyl) 4,7,7-trimethyl-3-oxo-2-oxabicyclo[2.2.1]heptane-1-carboxylate, ((*S*)-h) and (1*S*,4*R*)-((*R*)-1-(2-Bromophenyl)-2,2,2-trifluoro-1-phenylethyl) 4,7,7-trimethyl-3-oxo-2-oxabicyclo[2.2.1]heptane-1-carboxylate, ((*R*)-h). Potassium hydride (30%, 25 mmol) was added in a solution of 1-(2-bromophenyl)-2,2,2-trifluoro-1-phenylethanol (racemic-**g**, 7.5 g, 22.7 mmol) in anhydrous dichloromethane (125 mL) at 0 ^{o}C. After no bubbling was observed, (1*S*)-camphanic chloride (12.30 g, 56.75 mmol) was added. After stirring the reaction mixture at room temperature for 16 h, the mixture was chilled at 0 ^{o}C and then quenched by addition of water. The mixture was washed with water, saturated sodium bicarbonate and then brine (100 mL of each). The organic layer was then dried with magnesium sulfate, filtered and concentrated. The crude residue was purified with column chromatography (petroleum ether/ethyl acetate, 30:1). The (*S*)-**h**, [(1*S*,4*R*)-((*S*)-1-(2-bromophenyl)-2,2,2-trifluoro-1-phenylethyl) 4,7,7-trimethyl-3-oxo-2-oxabicyclo[2.2.1]heptane-1-carboxylate, eluted faster than the (*R*)-**h,** [(1*S*,4*R*)-((*R*)-1-(2-bromophenyl)-2,2,2-trifluoro-1-phenylethyl) 4,7,7-

trimethyl-3-oxo-2-oxabicyclo[2.2.1]heptane-1-carboxylate. (*S*)-**h** is (3.82 g, 7.48 mmol, 33% yield). ^{1}H NMR (400 MHz, $CDCl_3$, δ): 1.02 (s, 3H), 1.10 (s, 3H), 1.15 (s, 3H), 1.70–1.74 (m, 1H), 1.90–1.97 (m, 1H), 2.12–2.18 (m, 1H), 2.38–2.43 (m, 1H), 7.26–7.30 (m, 1H), 7.32–7.45 (m, 5H), 7.49 (t, *J* = 8 Hz, 1H), 7.55 (d, *J* = 8 Hz, 1H), 7.88 (d, *J* = 8 Hz, 1H). ^{13}C NMR (100 MHz, $CDCl_3$, δ): 9.71, 16.39, 16.48, 29.05, 31.27, 54.99, 55.09, 85.80 (q, *J* = 29 Hz), 91.18, 122.39, 123.74 (q, *J* = 286 Hz), 127.02, 127.90, 128.24, 128.91 (q, *J* = 4 Hz), 129.08, 130.27, 134.62, 135.08, 135.80, 164.03, 178.46. ^{19}F NMR (376 MHz, $CDCl_3$, δ): –68.77 (s). (*R*)-**h** is (3.58 g, 7.02 mmol, 31% yield). ^{1}H NMR (400 MHz, $CDCl_3$, δ): 1.04 (s, 3H), 1.15 (s, 3H), 1.24 (s, 3H), 1.74–1.80 (m, 1H), 1.98–2.05 (m, 1H), 2.22–2.29 (m, 1H), 2.61–2.68 (m, 1H), 7.25–7.29 (m, 1H), 7.31–7.39 (m, 5H), 7.48 (t, *J* = 8 Hz, 1H), 7.55 (d, *J* = 4 Hz, 1H), 7.88 (d, *J* = 8 Hz, 1H).^{13}C NMR ($CDCl_3$, δ): 9.65, 16.65, 16.89, 29.18, 31.56, 54.41, 54.88, 86.18 (q, *J* = 29 Hz), 91.10, 122.36, 123.81 (q, *J* = 289 Hz), 127.07, 127.99, 128.03, 129.10 (q, *J* = 4 Hz), 129.16, 130.36, 134.60, 135.27, 135.81, 163.29, 177.74. ^{19}F NMR (376 MHz, $CDCl_3$, δ): –68.57 (s). Crystals of the (*S*)-**h** were formed by slow evaporation of the product from diethyl ether solution. Crystals are good enough for X-ray crystal crystallography.

(*S*)-1-(2-Bromophenyl)-2,2,2-trifluoro-1-phenylethanol, ((*S*)-g). Sodium hydroxide (0.59 g, 14.89 mmol) was added to a solution of [(1*S*,4*R*)-((*S*)-1-(2-bromophenyl)-2,2,2-trifluoro-1-phenylethyl) 4,7,7-trimethyl-3-oxo -2-oxabicyclo[2.2.1]heptane-1-carboxylate], (*S*)-**h**, (3.80 g, 7.45 mmol) in tetrahydrofuran (82 mL) and methanol (32 mL). After stirring the reaction mixture for 3 h, the solvent was removed. The

residue was then purified with column chromatography (petroleum ether/ethyl acetate, 30:1) to yield (*S*)-1-(2-bromophenyl)-2,2,2-trifluoro-1-phenylethanol ((*S*)-**g**, 2.31 g, 6.99 mmol, 94% yield). ^{1}H NMR (400 MHz, $CDCl_3$, δ): 3.96 (s, 1H), 7.25 (t, *J* = 8 Hz, 1H), 7.31–7.39 (m, 5H), 7.42 (t, *J* = 4 Hz, 1H), 7.59 (d, *J* = 4 Hz, 1H), 7.82 (d, *J* = 4 Hz, 1H). ^{13}C NMR (100 MHz, $CDCl_3$, δ): 81.10 (q, *J* = 29 Hz), 122.82, 124.54 (q, *J* = 284 Hz), 127.16, 127.89, 127.94, 128.66, 129.25 (q, *J* = 4 Hz), 130.21, 135.92, 137.45, 137.96. ^{19}F NMR (376 MHz, $CDCl_3$, δ): –72.46 (s).

(1*S*,1'*S*)-1,1'-(Biphenyl-2,2'-diyl)bis(2,2,2-trifluoro-1-phenylethanol), (6). (*S*)-1-(2-bromophenyl)-2,2,2-trifluoro-1-phenylethanol, (*S*)-**g**, (2.00 g, 6.06 mmol) was added to a suspension of bis(1,5-cyclooctadiene)nickel(0) (0.95 g, 3.45 mmol) in 2.0 mL anhydrous dimethylformamide under nitrogen. The mixture was heated at 90 ^{o}C for 16 h. After cooling to room temperature, the reaction was quenched by addition of 5% aqueous hydrochloric acid and then extracted with diethyl ether. The extract was dried with anhydrous magnesium sulfate and purified with column chromatography (petroleum ether/ dichloromethane, 20:1) to yield **6** (1.00 g, 2.00 mmol, 66%). ^{1}H NMR (400 MHz, $CDCl_3$, δ): 4.35 (s, 2H), 5.73 (d, *J* = 4 Hz, 2H), 6.76 (t, *J* = 8 Hz, 2H), 7.08 (d, *J* = 8 Hz, 4H), 7.20–7.32 (m, 8H), 7.79 (d, *J* = 8 Hz, 2H). ^{13}C NMR (100 MHz, $CDCl_3$, δ): 80.53 (q, *J* = 28 Hz), 124.94 (q, J = 285 Hz), 126.17 (q, J = 5 Hz), 126.58, 127.05, 127.16, 127.84, 128.46, 132.00, 135.17, 138.70, 140.31. ^{19}F NMR (376 MHz, $CDCl_3$, δ): –72.89 (s). Crystals of **6** were formed by slow evaporation of the products from diethyl ether solution.

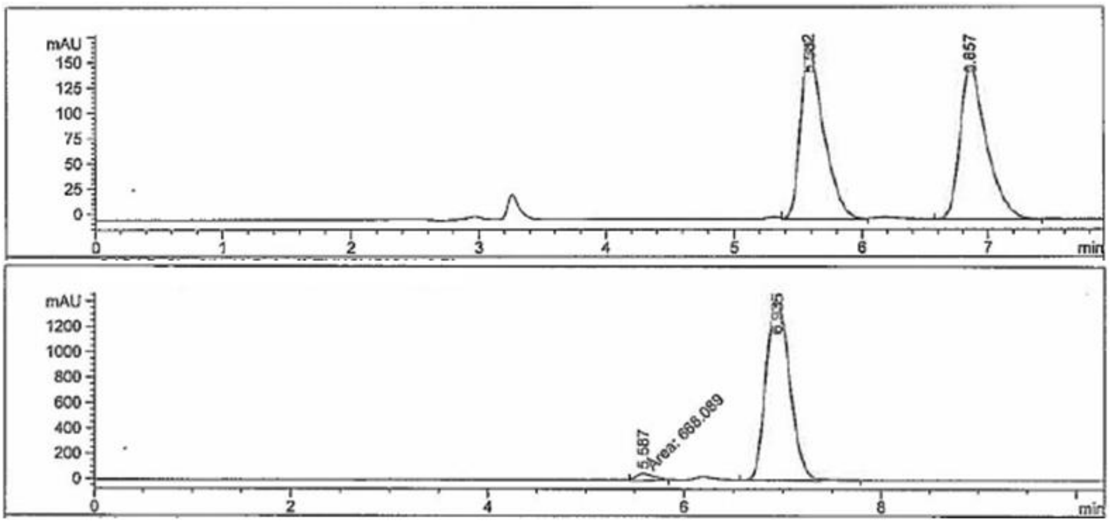

Figure S1. HPLC chromatograms of (top) a racemic mixture of **b** and (bottom) (*S*)-**b**. CHRIALCEL ® OD, 80:20 Hexane/iso-propanol, 1mL/min

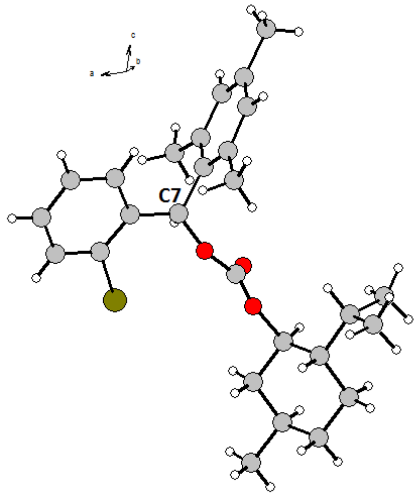

Figure S2. X-ray crystallography of (*R*)-**e,** (*R*)-(2-bromophenyl)(mesityl)methyl (1*R*,2*S*,5*R*)-2-isopropyl-5-methylcyclohexyl carbonate showing *R*-configuration at C7.

Table S1. Crystal data and structure refinement of (*R*)-**e**, (*R*)-(2-bromophenyl)(mesityl)methyl(1*R*,2*S*,5*R*)-2-isopropyl-5-methyl cyclohexyl carbonate

Empirical formula	C27 H35 Br O3
Formula weight	487.46
Temperature	296(2) K
Wavelength	0.71073 Å
Crystal system, space group	Monoclinic, P 21
Unit cell dimensions	a = 8.8603(3) Å alpha = 90 º
	b = 12.4438(4) Å beta = 110.4517(16) º
	c = 12.6231(3) Å gamma = 90 º
Volume	1304.04(7) A^3
Z	2
Absorption coefficient	1.600 mm^{-1}
F(000)	512
Crystal size	0.56 x 0.52 x 0.20 mm
θ range for data collection	2.950 - 26.724
Limiting indices	-11 ≤ h ≤ 11, -15 ≤ k ≤ 15, -15 ≤ l ≤ 15
Reflections collected / unique	23467 / 5502
Completeness to θ	99.8%
Absorption correction	Multi-scan
Max. and min. transmission	0.7403 / 0.4678
Refinement method	Full-matrix least-squares treatment on F^2
Data / restraints / parameters	5502 / 1 / 286
Goodness-of-fit on F^2	1.030
Final R indices [I>2sigma(I)]	R1 = 0.0398, wR2 = 0.0668

R indices (all data)	R1 = 0.0797, wR2 = 0.0782
Absolute structure parameter	0.040(5)
Largest diff. peak and hole	0.212 and -0.251 eÅ^{-3}

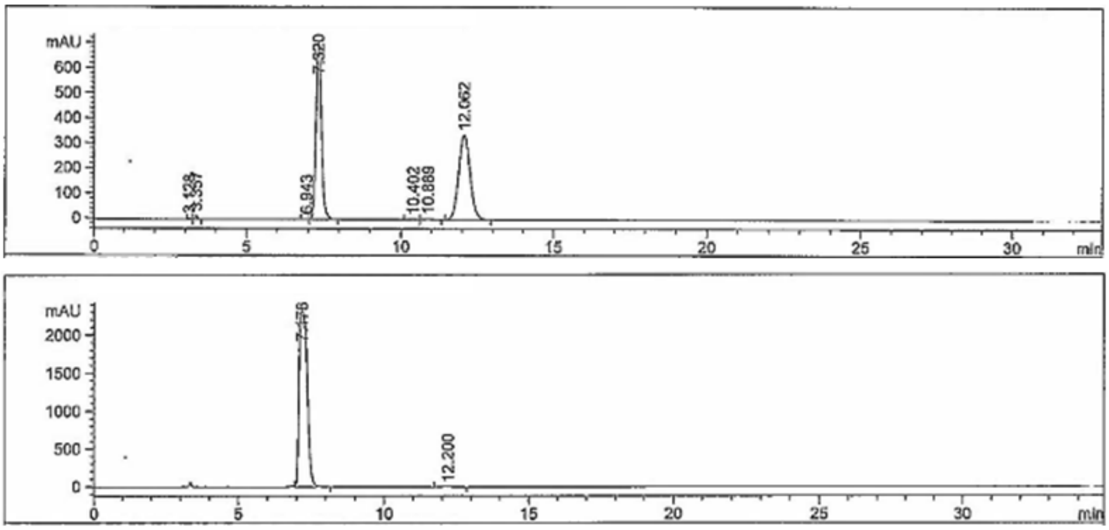

Figure S3. HPLC chromatograms of (top) a racemic mixture of **d** and (bottom) (*R*)-**d**. CHIRALCEL ® OJ-H, 90:10 Hexane/iso-propanol, 1mL/min

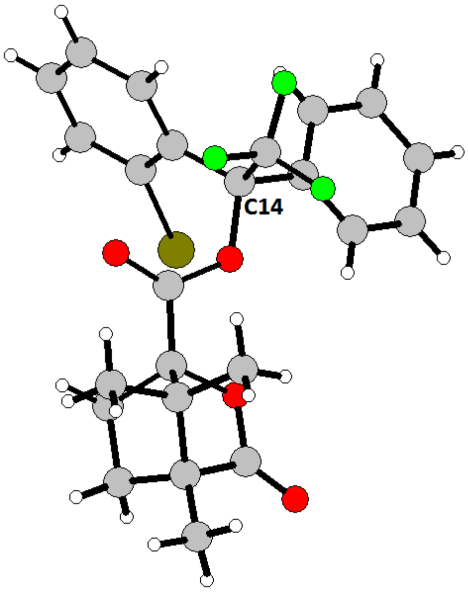

Figure S4. X-ray crystallography of (*S*)-**h**, [(1*S*,4*R*)-((*S*)-1-(2-bromophenyl)-2,2,2-trifluoro-1-phenylethyl) 4,7,7-trimethyl-3-oxo-2-oxabicyclo[2.2.1]heptane-1-carboxylate, showing *S*-configuration at C14.

Table S2. Crystal data and structure refinement of (*S*)-**h**, [(1*S*,4*R*)-((*S*)-1-(2-bromophenyl)-2,2,2- trifluoro-1-phenylethyl) 4,7,7-trimethyl-3-oxo-2-oxabicyclo[2.2.1]heptane-1- carboxylate

Empirical formula	C24 H22 Br F3 O4
Formula weight	511.33
Temperature	298(2) K
Wavelength	0.71073 Å
Crystal system, space group	Orthorhombic, P 21 21 21
Unit cell dimensions	a = 7.4357(4) Å alpha = 90 º
	b = 11.6828(7) Å beta = 90 º
	c = 26.2324(16) Å gamma = 90 º
Volume	2278.8(2) A^3
Z	4
Absorption coefficient	1.856mm^{-1}
F(000)	1040
Crystal size	0.50 x 0.46 x 0.28 mm
θ range for data collection	2.843 - 27.482
Limiting indices	-9 ≤ h ≤ 9, -15 ≤ k ≤ 15, -34 ≤ l ≤ 33
Reflections collected / unique	17657 / 5087
Completeness to θ	95.3%
Absorption correction	Multi-scan
Max. and min. transmission	0.6263 / 0.4359
Refinement method	full-matrix least-squares treatment on F^2
Data / restraints / parameters	5087 / 0 / 293
Goodness-of-fit on F^2	0.978
Final R indices [I>2sigma(I)]	R1 = 0.0362, wR2 = 0.0954

R indices (all data)	R1 = 0.0751, wR2 = 0.1192
Absolute structure parameter	0.024(5)
Largest diff. peak and hole	0.481 and -0.918 eÅ^{-3}

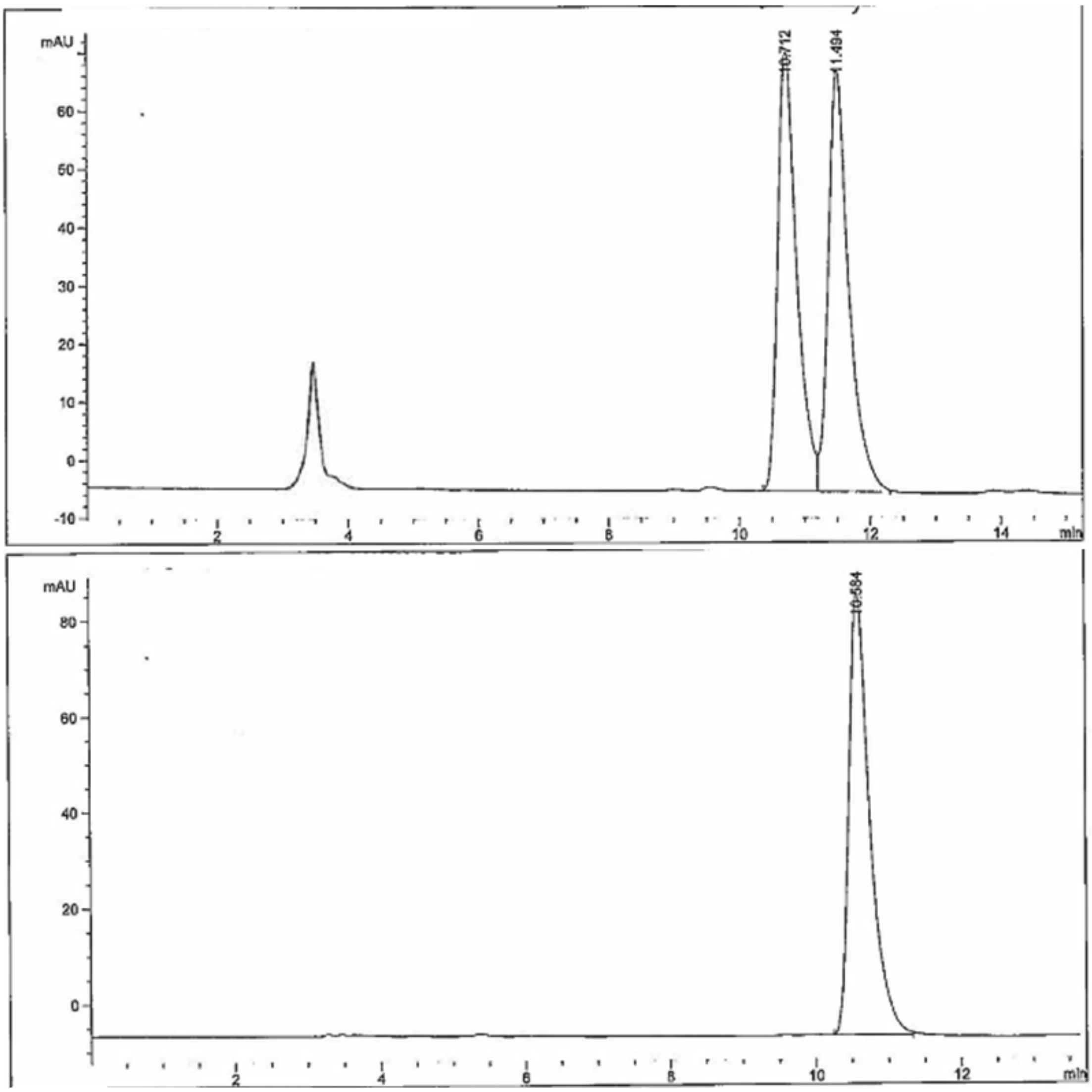

Figure S5. HPLC chromatograms of (top) a racemic mixture of **g** and (bottom) (*S*)-**g**. CHIRALCEL ® AD-H, 98:2 hexane/isopropanol, 1mL/min

Table S3. Crystal data and structure refinement of **3** and **6**

	**3**	**6**
Empirical formula	C26H22O2	C28H20F6O2
Formula weight	366.44	502.44
Temperature	296(2)	293(2)
Wavelength	0.71073	0.71073
Crystal system, space group	Monoclinic, P2	Monoclinic, P21
Unit cell dimensions	a=9.3562(3)	a=10.7357(16)
	b=11.6980(4)	b=13.1589(18)
	c=9.4294(4)	c=16.582(2)
	alpha=90	alpha=90
	beta=106.6234(21)	beta=94.6136(41)
	gamma=90	gamma=90
Volume	988.912(63)	2335.0(6)
Z	2	4
Absorption coefficient	0.076 mm^{-1}	0.121 mm^{-1}
F(000)	388	1032
Crystal size	0.18 x 0.24 x 0.38	0.20 x 0.26 x 0.40
θ range for data collection	1.741 - 27.631	2.453 - 27.559
Limiting indices	-12 ≤ h ≤ 12	-12 ≤ h ≤ 13
	-14 ≤ k ≤ 15	-17 ≤ k ≤ 17
	-12 ≤ l ≤ 12	-21 ≤ l ≤ 21
Reflections collected / unique	22038 / 4430	51855 / 10729
Completeness to θ	99.4%	99.7%
Absorption correction	Multi-scan	
Max. and min. transmission	0.9864 / 0.9716	0.9761 / 0.9531
Refinement method	Full-matrix least-squares treatment on F^2	
Data / restraints / parameters	4430 / 1 / 254	10729 / 1 / 661
Goodness-of-fit on F^2	1.093	1.039
Final R indices [I>2σ(I)]	R1 = 0.0592	R1 = 0.0421

	wR2 = 0.1576	wR2 = 0.0900
R indices (all data)	R1 = 0.0734	R1 = 0.0622
	wR2 = 0.1656	wR2 = 0.0987
Absolute structure parameter	-1.1(5)	0.12(11)
Largest diff. peak and hole	0.209 and -0.203 eÅ^{-3}	0.138 and -0.165 eÅ^{-3}

General procedure for catalytic Hetero-Diels–Alder reactions

To a solution of the catalyst (0.1 mmol) and aldehyde (1.0 mmol) in toluene (0.5 mL) at –20 ^{o}C was added *trans*-3-(tert-butyldimethylsilyloxy)-N,N-dimethyl-1,3-butadien-1-amine (0.5 mmol). The reaction was maintained at this temperature for 1 day. Then the reaction was cooled at –78°C, diluted with 1.0 mL of dichloromethane, treated with acetyl chloride (1.0 mmol). After stirring for 20 min, the crude was directly purified with column chromatography to afford the product.

2,3-Dihydro-2-phenyl-4*H*-pyran-4-one [4]

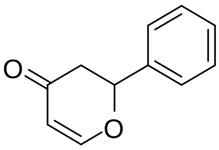

Isolated as a clear pale yellow oil: CHIRALCEL® OD-H, Hexane:*iso*-propanol 90:10, 1mL/min: t_R = 13.5 min (*S*) and 16.4 min (*R*). ^{1}H NMR (400 MHz, $CDCl_3$, δ): 2.68 (dd, *J* = 20, 4 Hz 1H), 2.92 (dd, *J* = 20, 16 Hz, 1H), 5.44 (dd, *J* = 16, 4 Hz, 1H), 5.54 (d, *J* = 4 Hz, 1H), 7.39–7.43 (m, 5H), 7.49 (d, *J* = 4Hz, 1H).

2,3-Dihydro-2-*t*-butyl-4*H*-pyran-4-one [5]

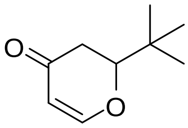

Isolated as a pale yellow oil: CHIRALCEL® OD-H, Hexane:*iso*-propanol 98:2, 1mL/min: t_R = 10.7 min and 11.6 min. ^{1}H NMR (400 MHz, $CDCl_3$, δ): 0.99 (s, 9H), 2.40 (dd, *J* = 16, 4 Hz, 1H), 2.49–2.57 (m, 1H), 4.03 (dd, *J* = 12, 4 Hz, 1H), 5.41 (d, *J* = 4 Hz, 1H), 7.42 (d, *J* = 8Hz, 1H).

2,3-Dihydro-2-isopropyl-4*H*-pyran-4-one [6]

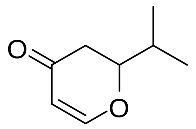

Isolated as a clear pale yellow oil: CHIRALCEL® OD-H, Hexane:*iso*-propanol 98:2, 1mL/min: t_R = 12.0 min (*S*) and 12.6 min (*R*), ^{1}H NMR (400 MHz, $CDCl_3$, δ): 0.99 (d, *J* = 4 Hz, 3H), 1.01 (d, *J* = 4 Hz, 3H), 1.96–2.01 (m, 1H), 2.39 (dd, *J* = 20, 4 Hz, 1H), 2.49–2.57 (m, 1H), 4.16–4.18 (m, 1H), 5.39 (d, *J* = 8 Hz, 1H), 7.38 (d, *J* = 8 Hz, 1H).

2,3-Dihydro-2-neopentyl-4*H*-pyran-4-one

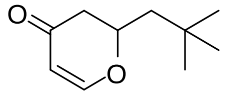

Isolated as a clear pale yellow oil: CHIRALCEL® OD-H, Hexane:*iso*-propanol 98:2, 1mL/min: t_R = 9.7 min and 10.2 min, ^{1}H NMR (400 MHz, $CDCl_3$, δ): 0.98 (s, 9H), 1.40 (dd, *J* = 15, 3 Hz, 1H), 1.86 (dd, *J* = 15, 8 Hz, 1H), 2.37 (dd, *J* = 17, 4 Hz, 1H), 2.53 (dd, *J* = 17, 14 Hz, 1H), 4.50–4.57 (m, 1H), 5.40 (d, *J* = 6 Hz, 1H), 7.34 (d, J = 6 Hz, 1H).

2,3-Dihydro-2-cyclohexyl-4*H*-pyran-4-one [4]

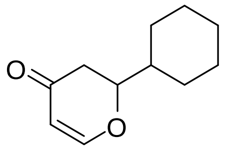

Isolated as a clear pale yellow oil: CHIRALCEL® OD-H, Hexane:*iso*-propanol 90:10, 1mL/min: t_R = 6.8 min (*S*) and 7.4 min (*R*), ^{1}H NMR (400 MHz, $CDCl_3$, δ): 1.0–1.32 (m, 5H), 1.66–1.72 (m, 3H), 1.78–1.83 (m, 2H), 1.84–1.93 (m, 1H), 2.40 (dd, *J* = 20, 4 Hz, 1H), 2.52–2.59 (m, 1H), 4.18–4.20 (m, 1H), 5.39 (d, *J* = 8 Hz, 1H), 7.38 (d, *J* = 8Hz, 1H).

2,3-Dihydro-2-benzyl-4*H*-pyran-4-one [7]

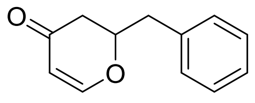

Isolated as a clear pale yellow oil: CHIRALCEL® OD-H, Hexane:*iso*-propanol 90:10, 1mL/min: t_R = 13.69 min and 14.91 min, ^{1}H NMR (400 MHz, $CDCl_3$, δ): 2.45 (dd, *J* = 20, 4 Hz, 1H), 2.55 (dd, *J* = 16, 4 Hz, 1H), 3.02 (dd, *J* = 20, 8 Hz, 1H), 3.14 (dd, *J* = 16, 4 Hz, 1H), 4.61–4.69 (m, 1H), 5.42 (d, *J* = 4Hz, 1H), 7.21–7.38 (m, 6H).

2,3-Dihydro-2-(naphthalen-2-yl)-4*H*-pyran-4-one

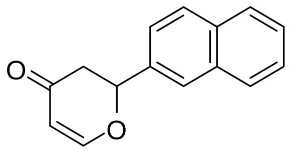

Isolated as a white solid: CHIRALCEL® OD-H, Hexane:*iso*-propanol 60:40, 1mL/min: t_R = 13.5 min and 21.3 min. ^1H NMR (400 MHz, $CDCl_3$, δ): 2.76 (dd, *J* = 16, 4 Hz, 1H), 3.01 (dd, *J* = 16, 12 Hz, 1H), 5.56-5.62 (m, 2H), 7.50-7.54 (m, 4H), 7.85-7.92 (m, 4H).

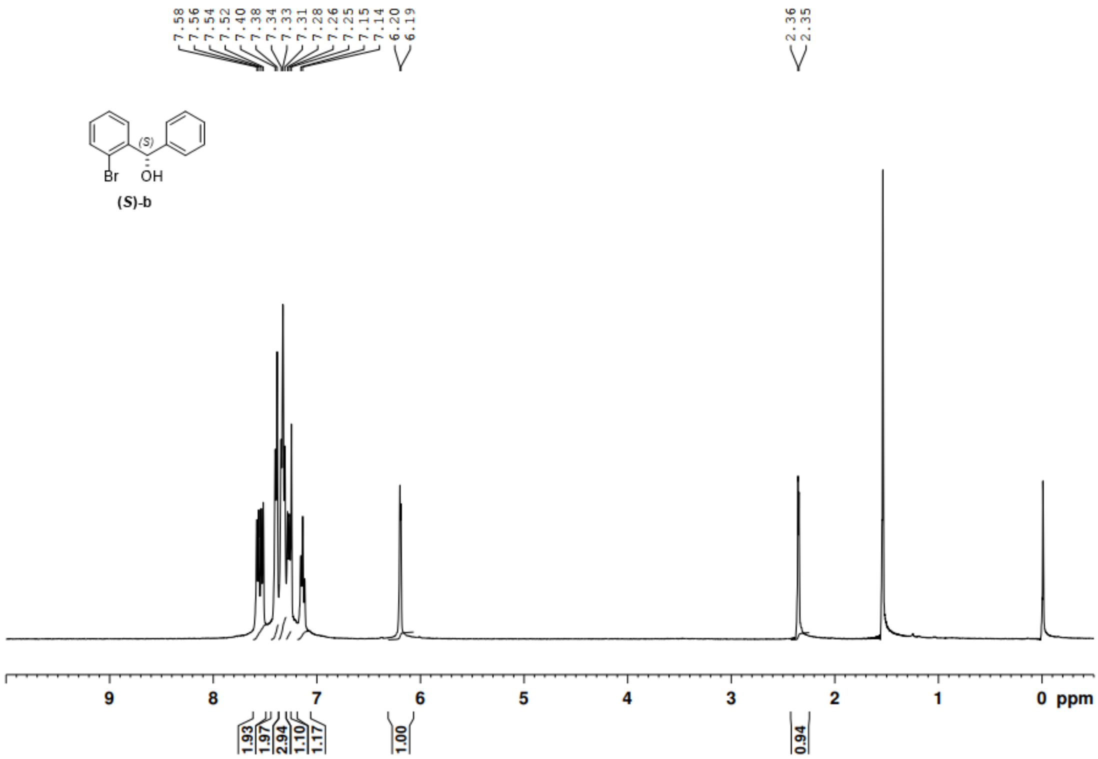

Figure S6. ^{1}H NMR of (*S*)-**b**

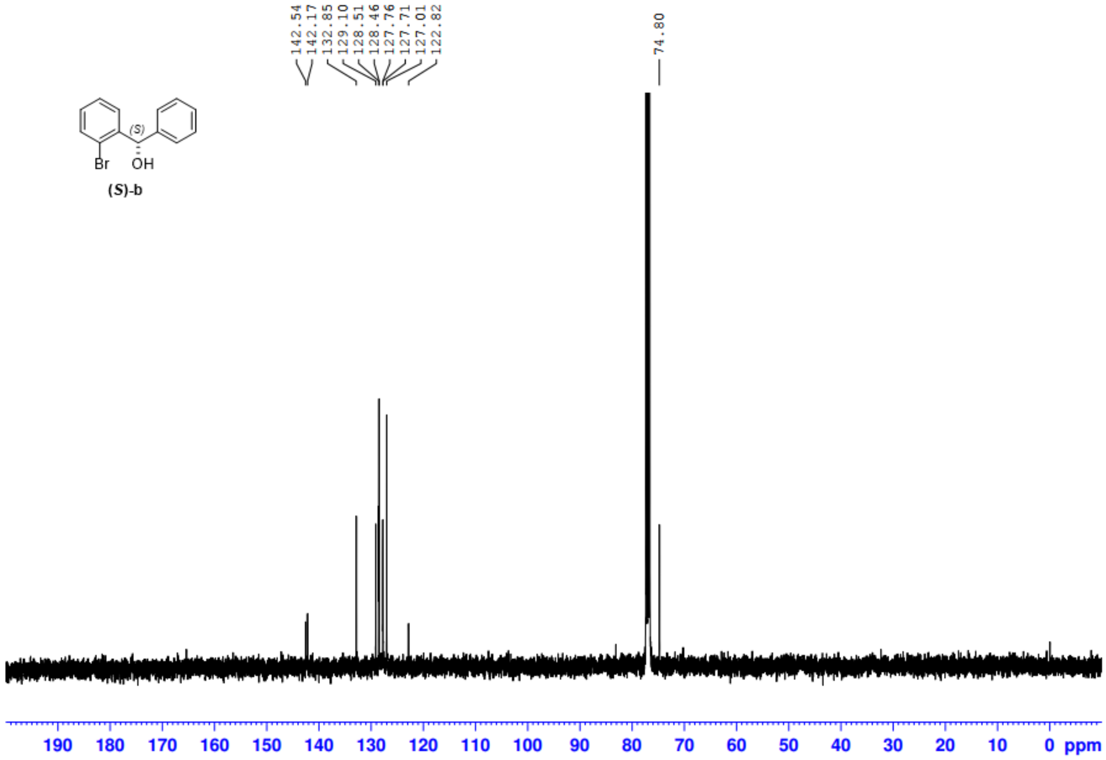

Figure S7. ^{13}C NMR of (*S*)-**b**

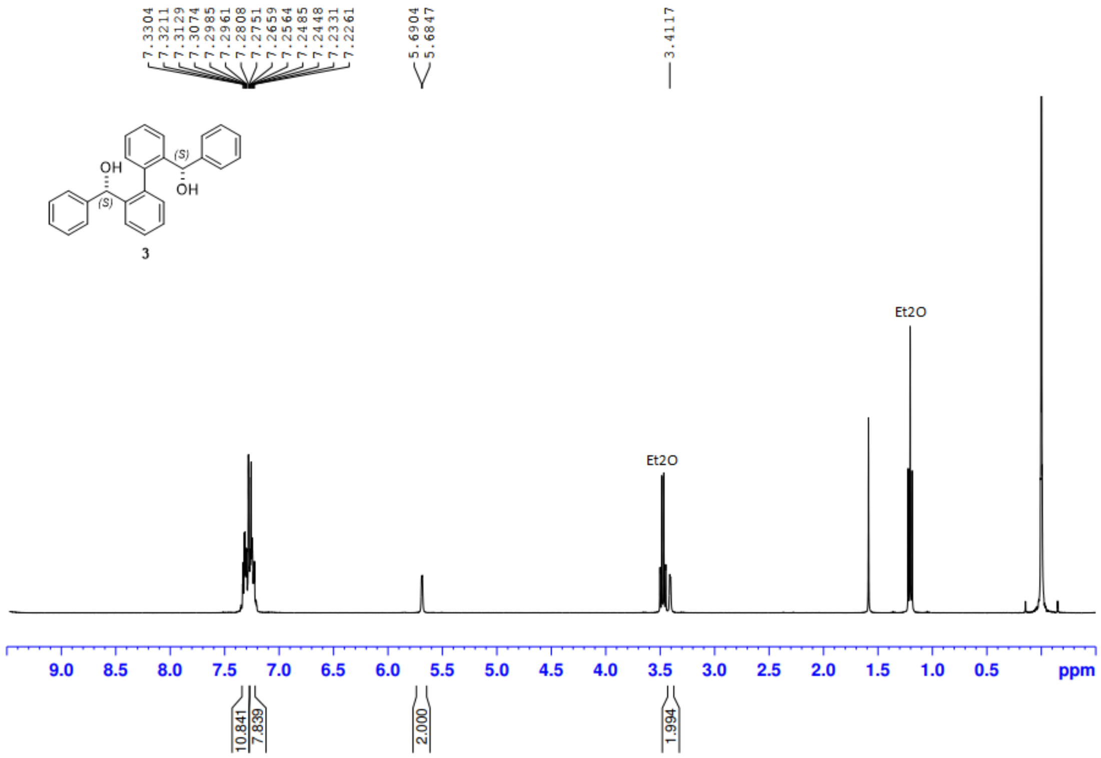

Figure S8. ^{1}H NMR of **3**

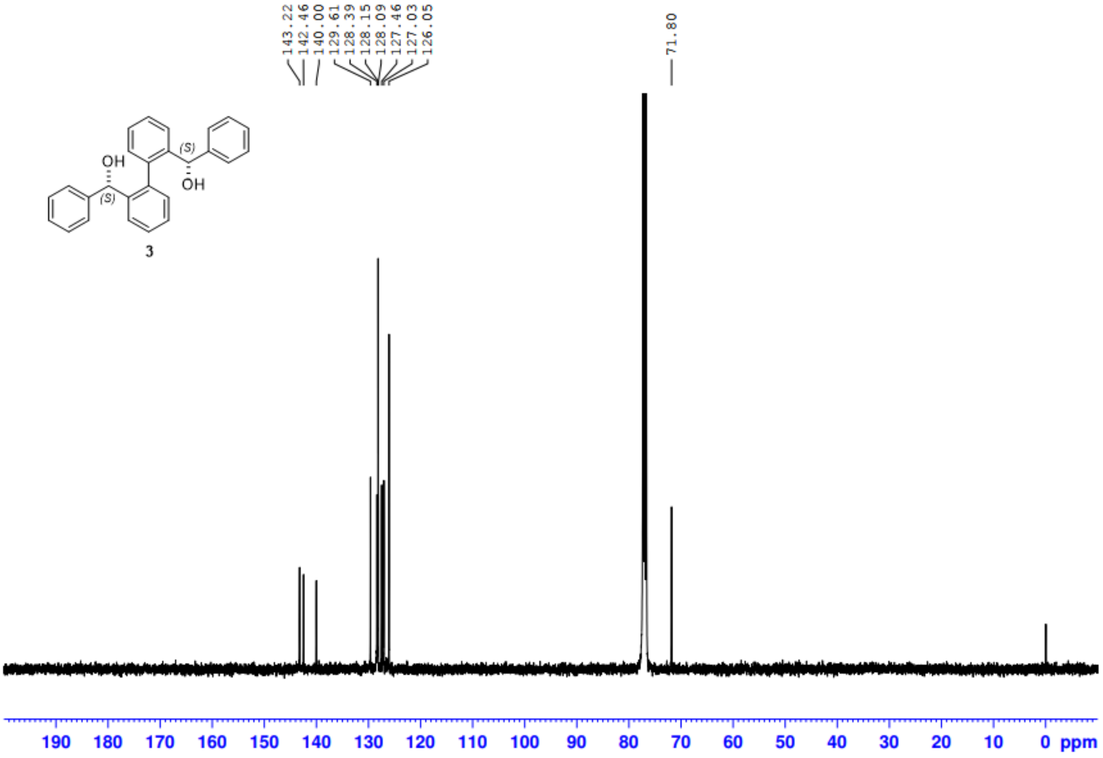

Figure S9. ^{13}C NMR of **3**

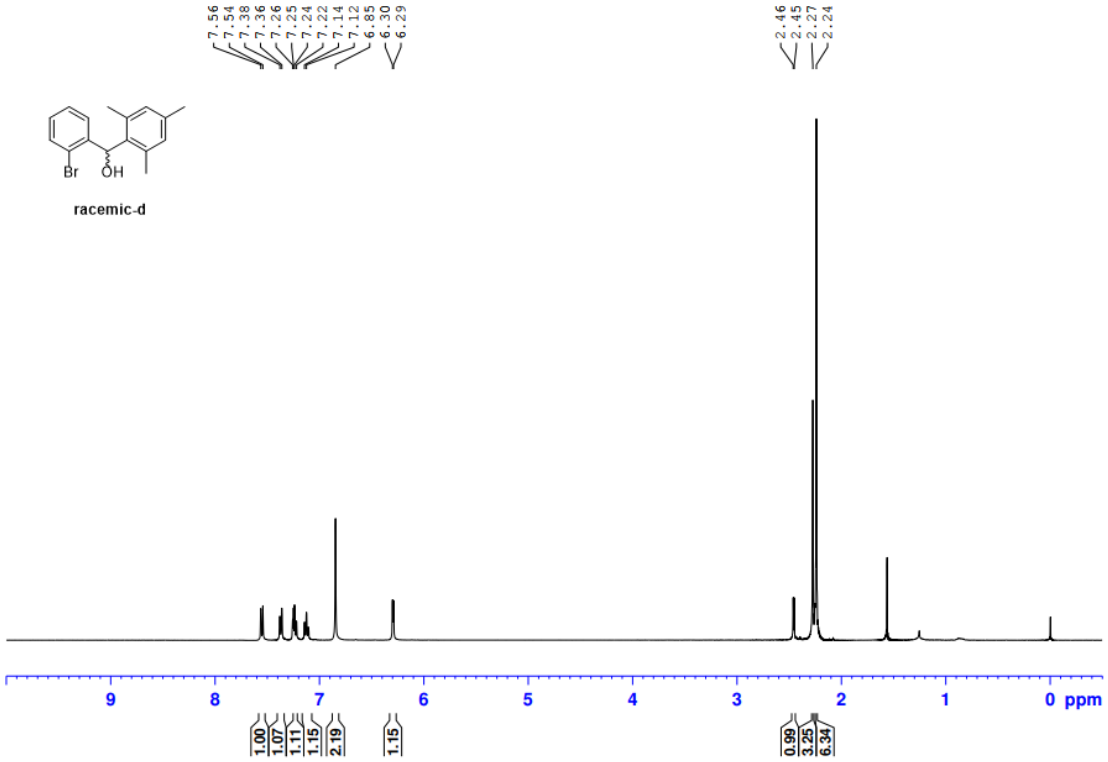

Figure S10. ^{1}H NMR of racemic-**d**

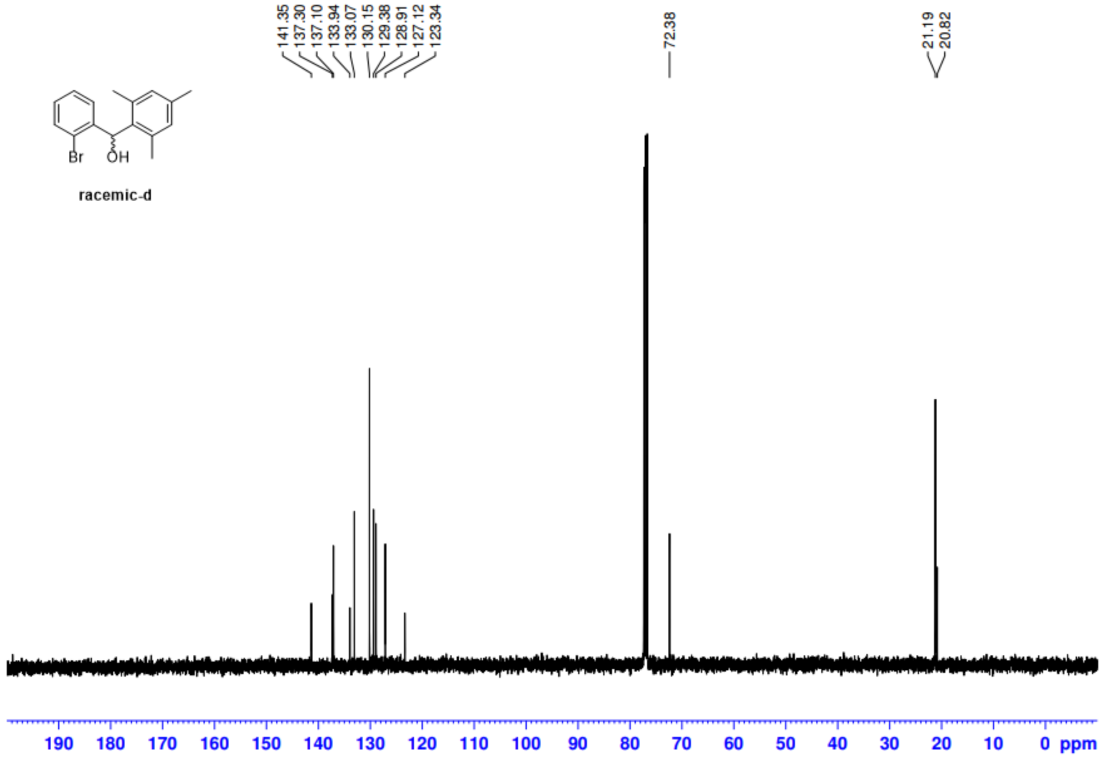

Figure S11. ^{13}C NMR of racemic-**d**

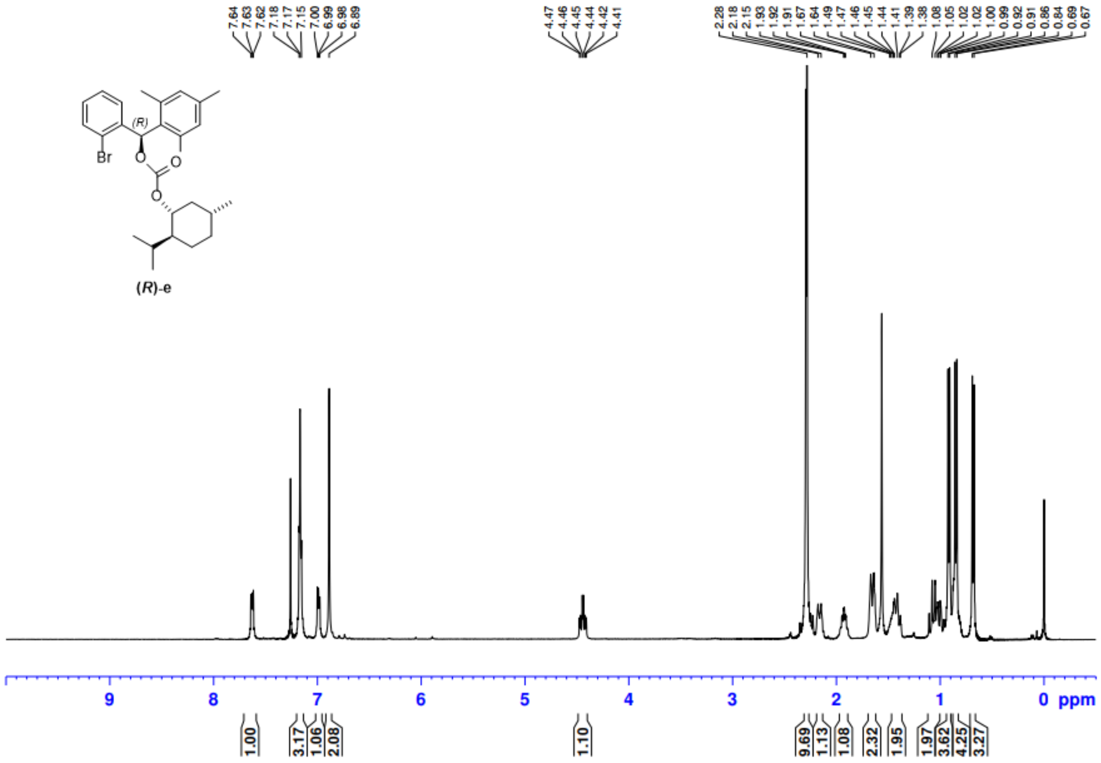

Figure S12. ^{1}H NMR of (*R*)-**e**

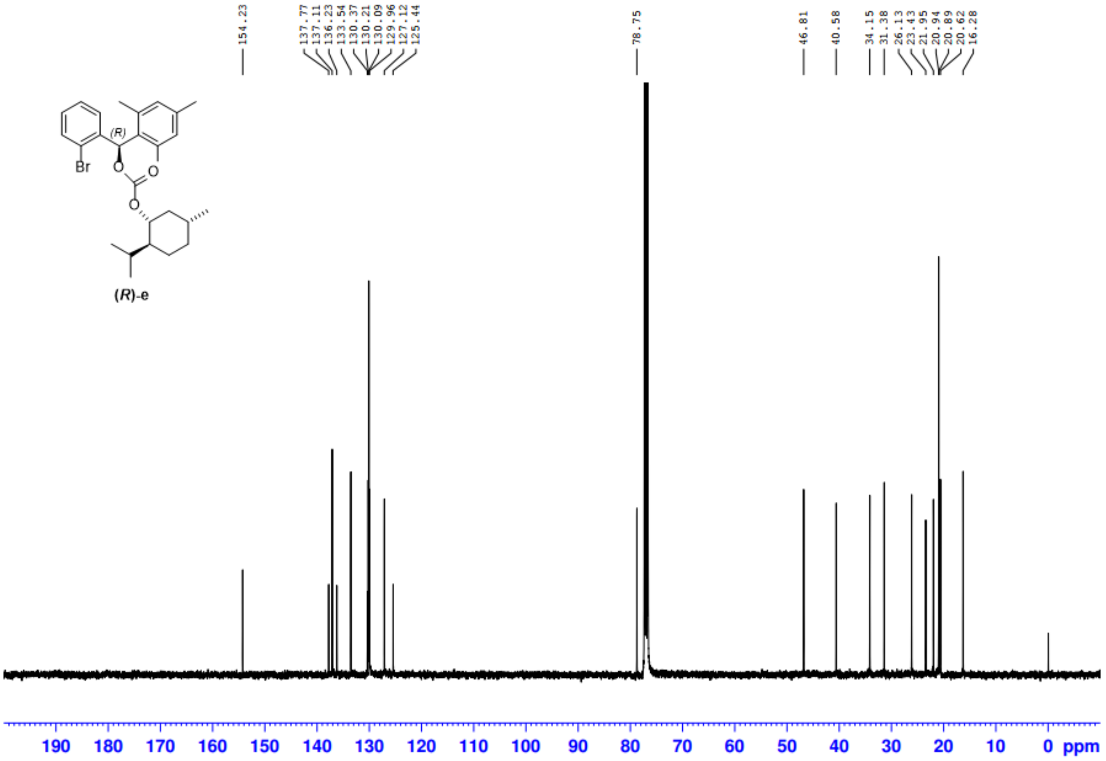

Figure S13. ^{13}C NMR of (*R*)-**e**

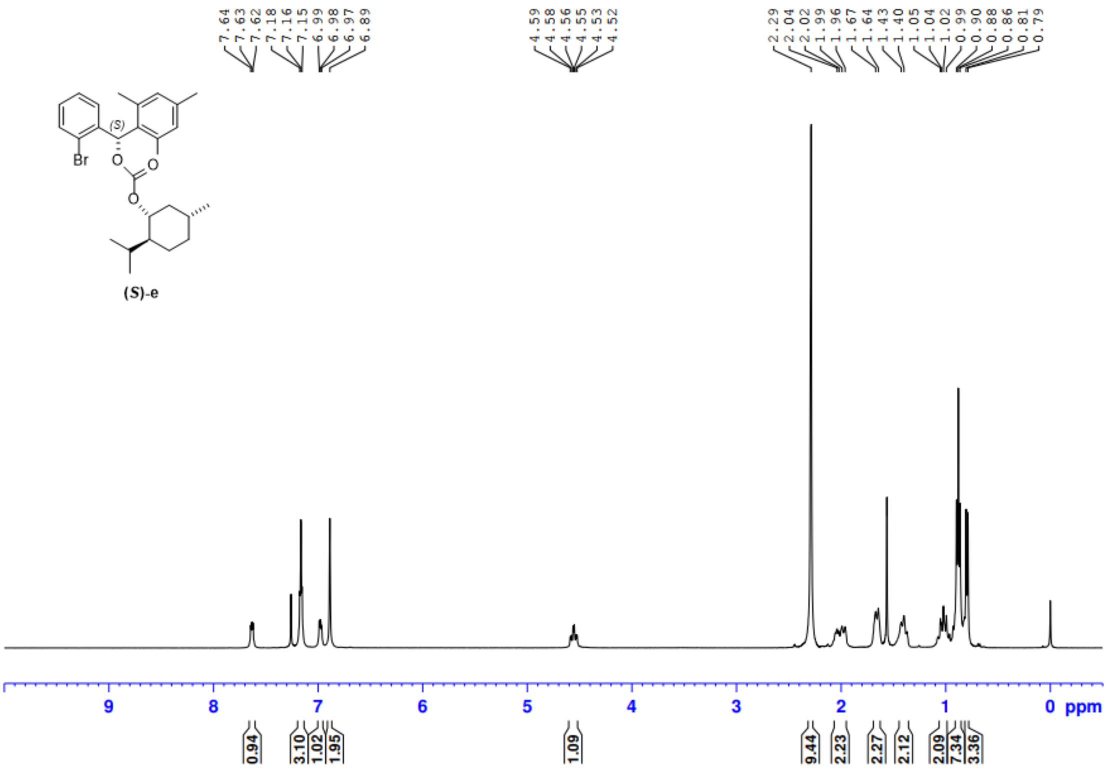

Figure S14. ^{1}H NMR of (*S*)-**e**

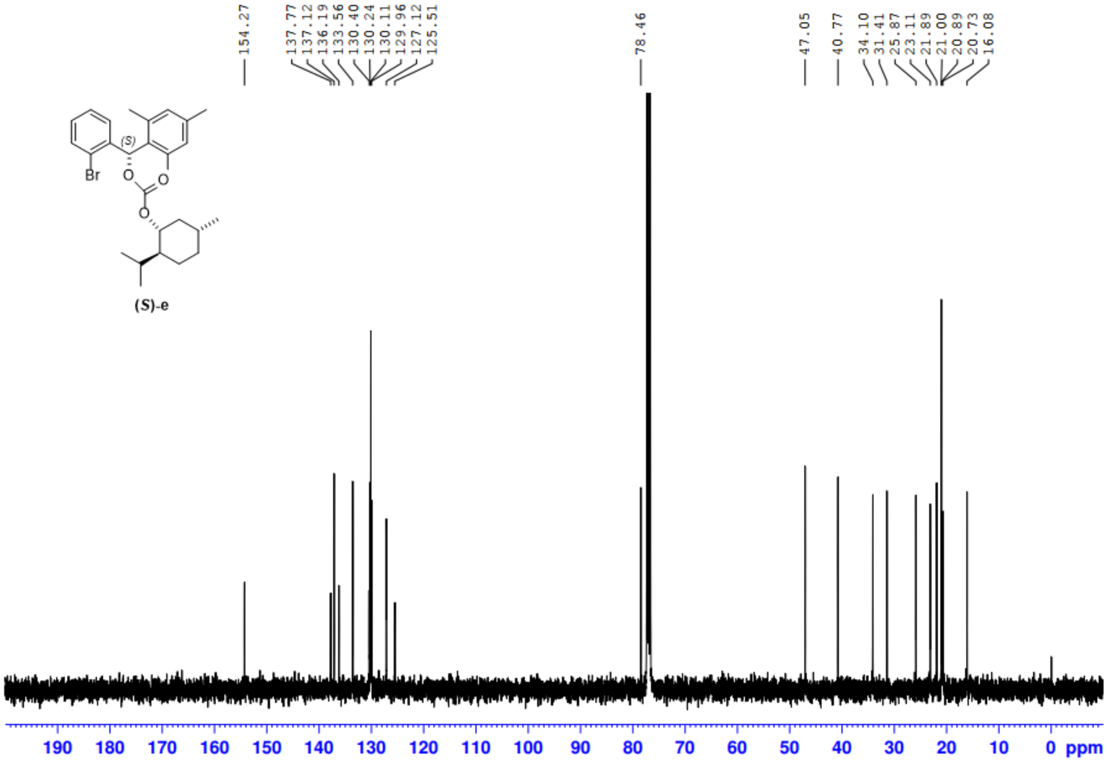

Figure S15. ^{13}C NMR of (*S*)-**e**

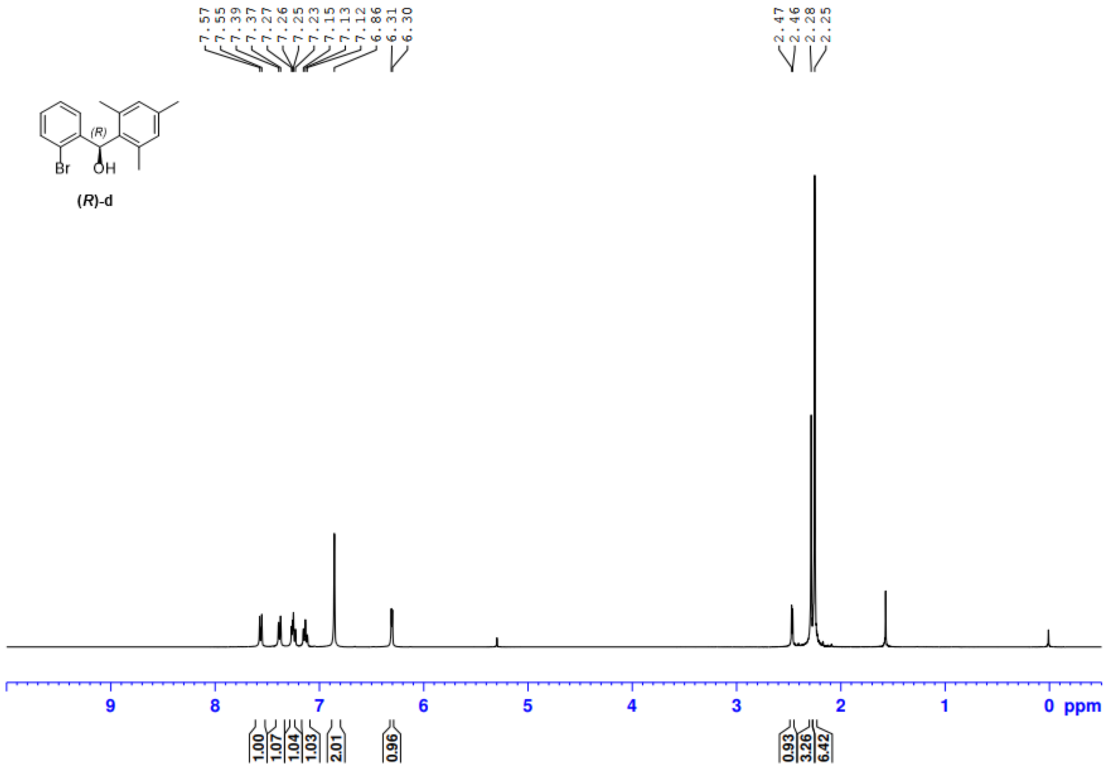

Figure S16. ^{1}H NMR of (*R*)-**d**

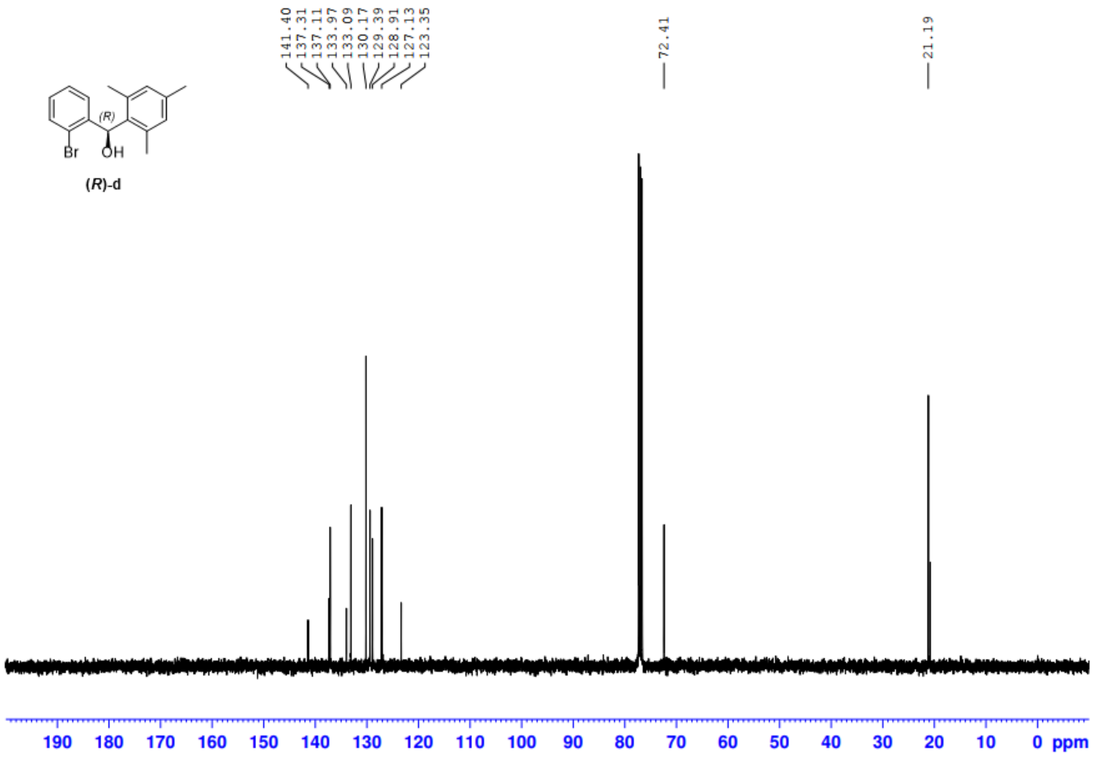

Figure S17. ^{13}C NMR of (*R*)-**d**

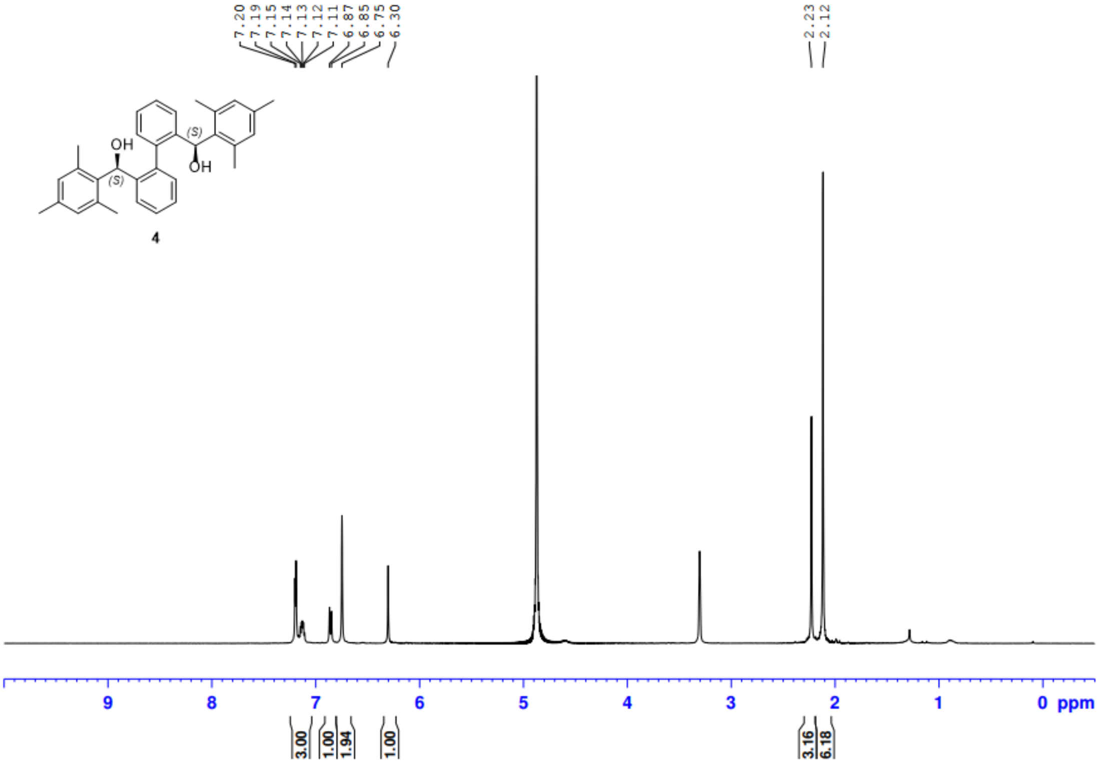

Figure S18. ^{1}H NMR of **4**

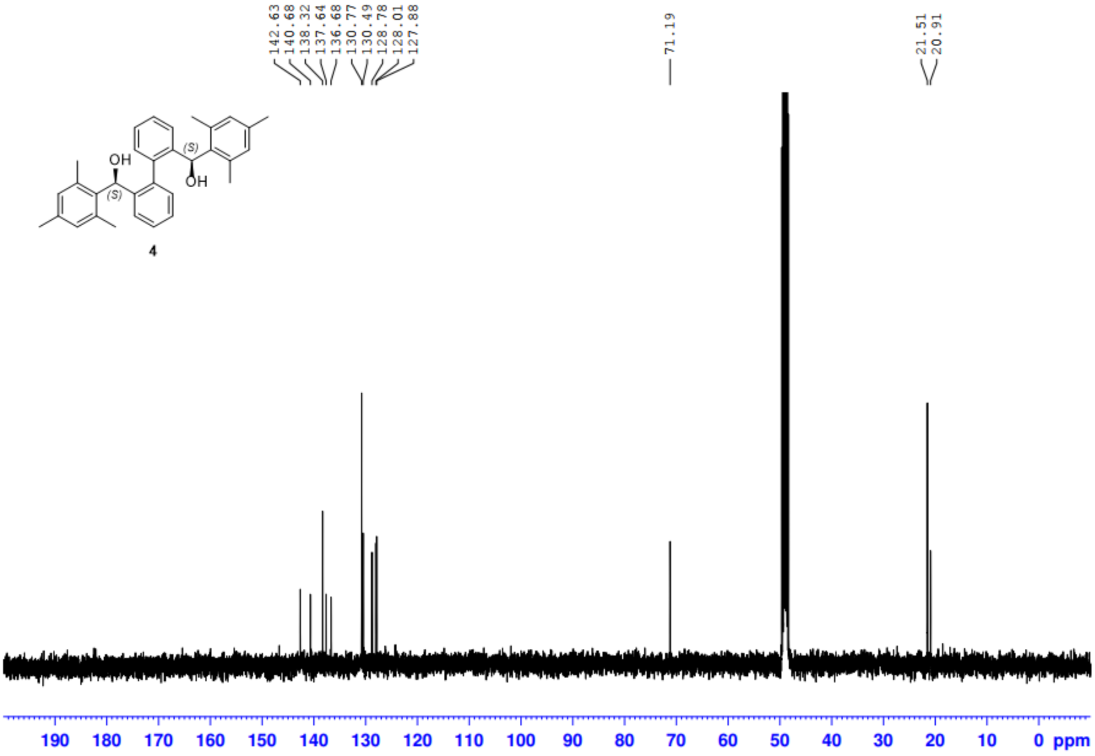

Figure S19. ^{13}C NMR of **4**

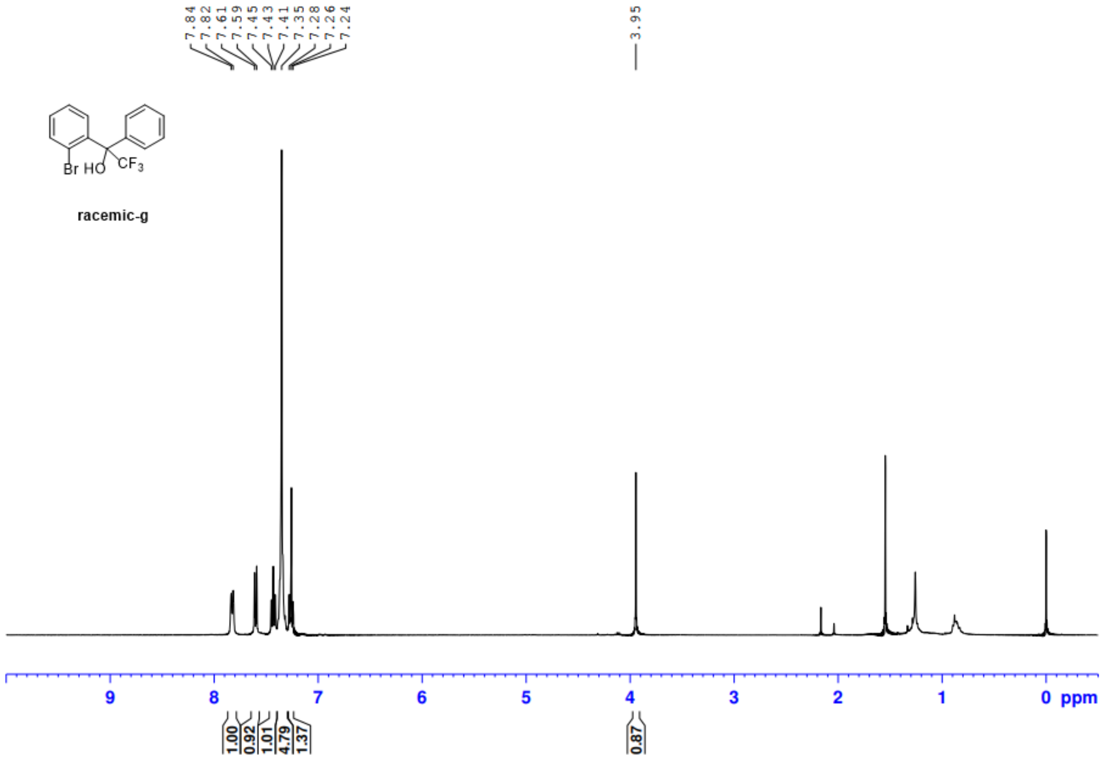

Figure S20. ^{1}H NMR of racemic-**g**

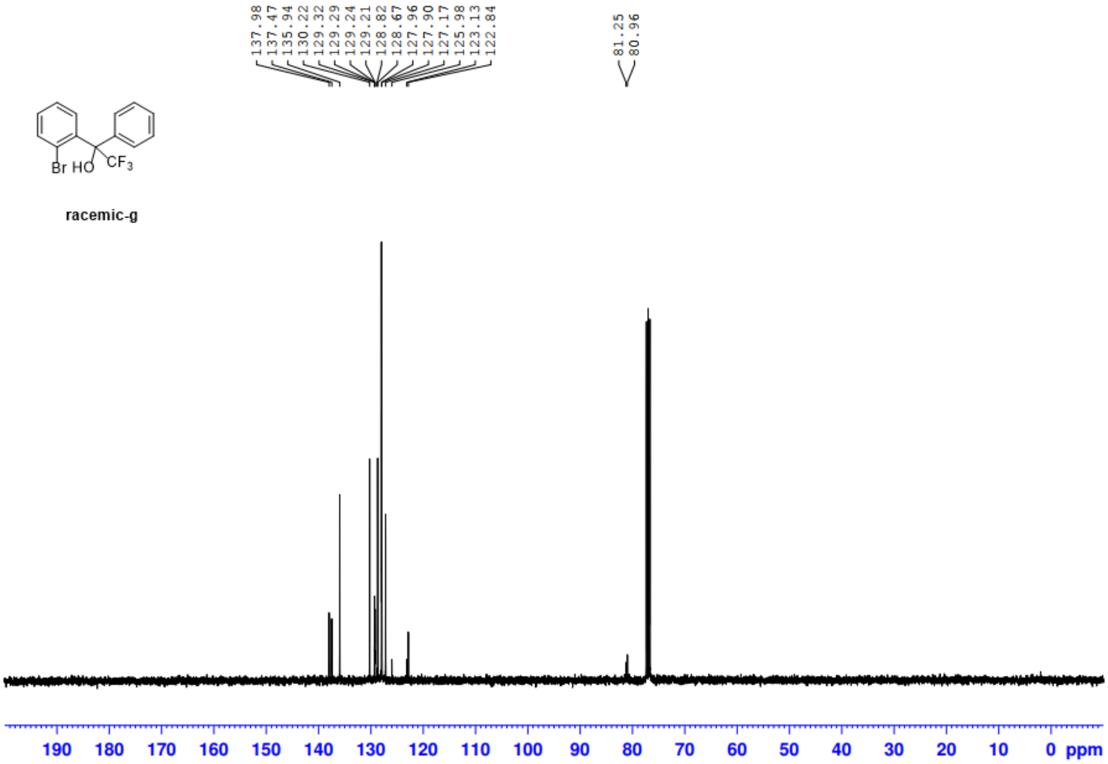

Figure S21. ^{13}C NMR of racemic-**g**

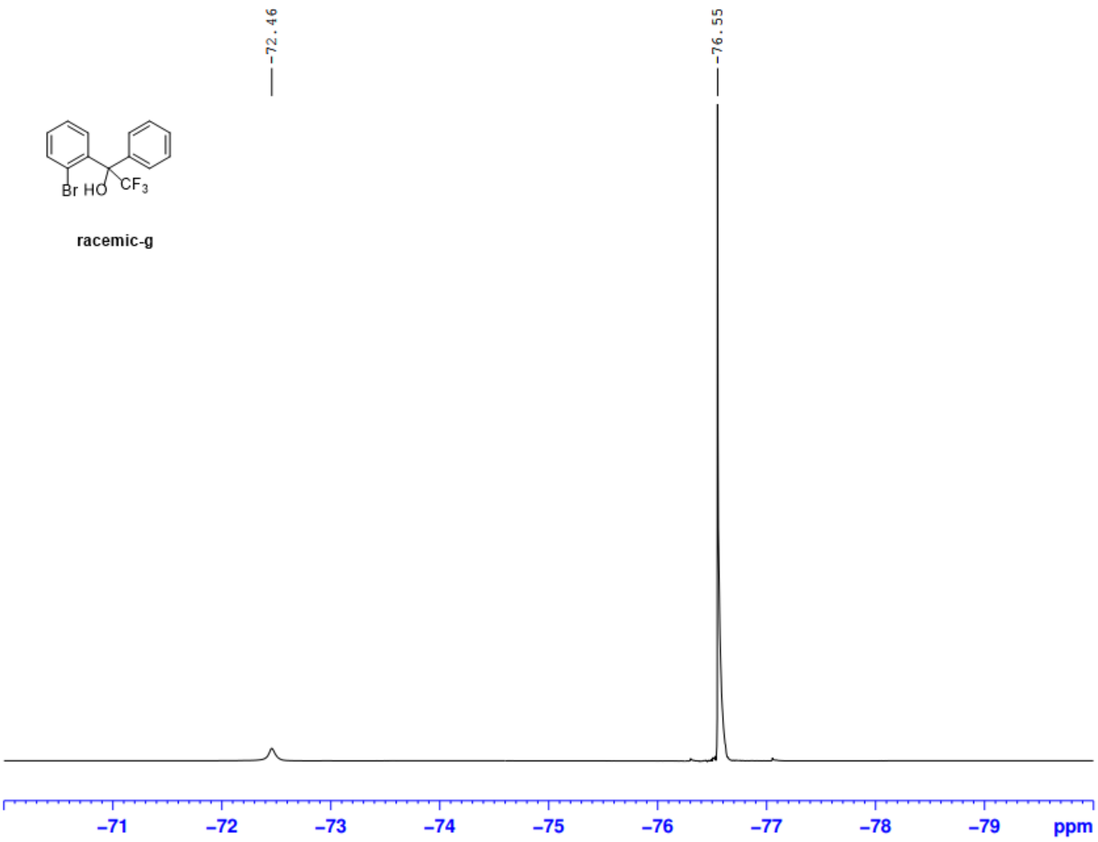

Figure S22. ^{19}F NMR of racemic-**g**

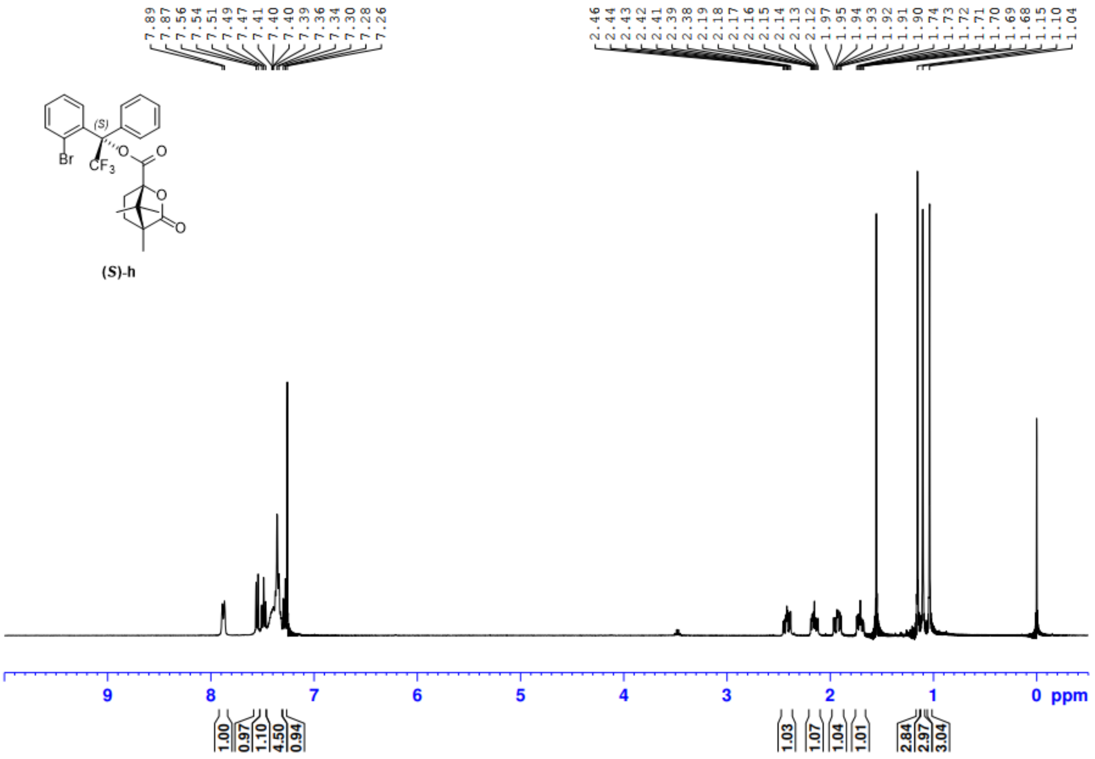

Figure S23. ^{1}H NMR of (*S*)-**h**

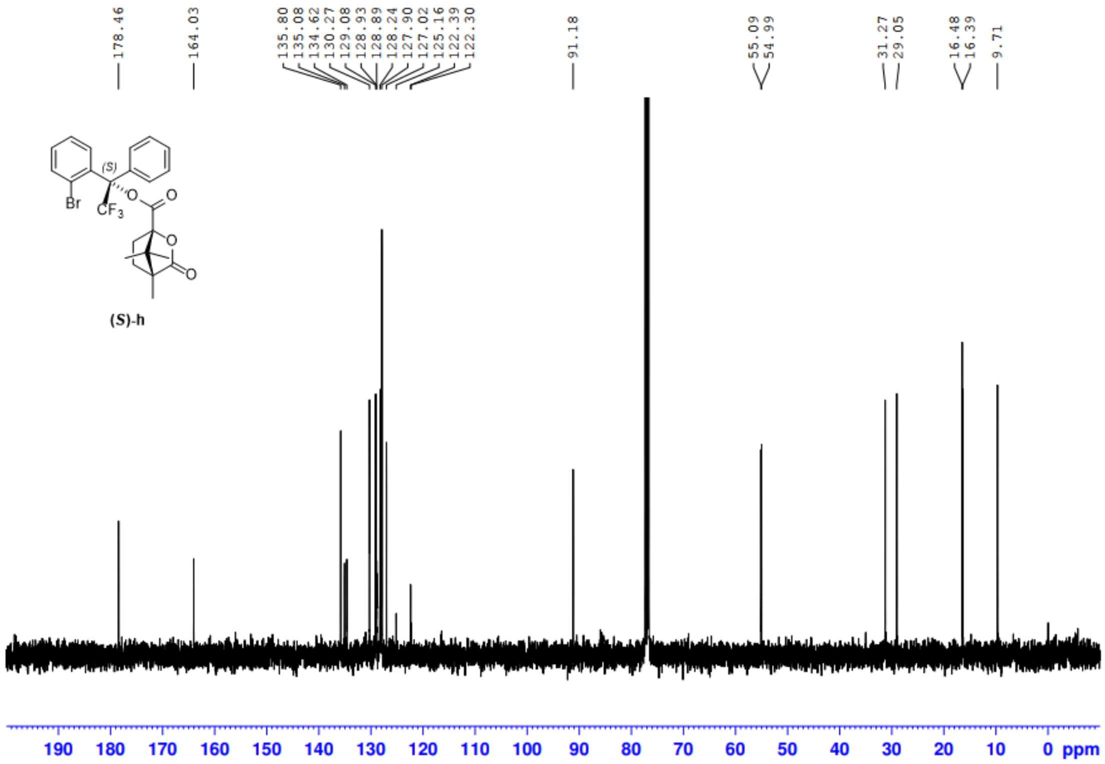

Figure S24. ^{13}C NMR of (*S*)-**h**

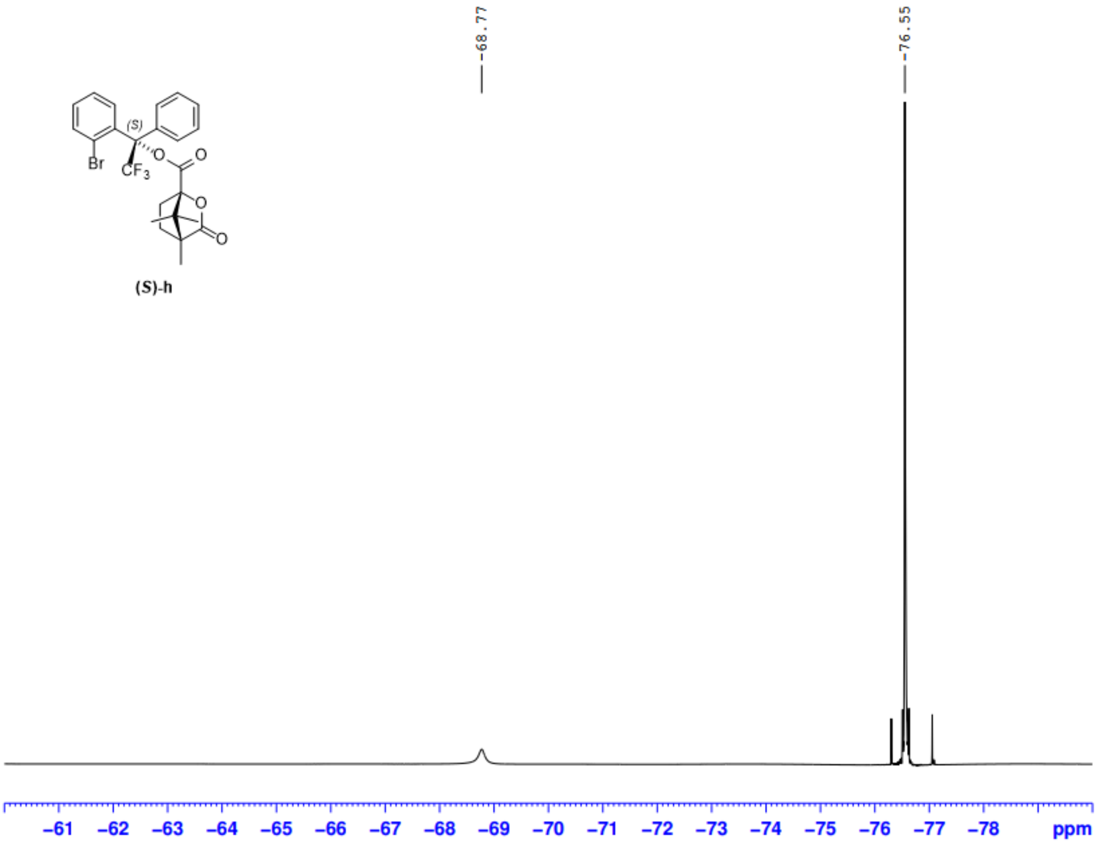

Figure S25. ^{19}F NMR of (*S*)-**h**

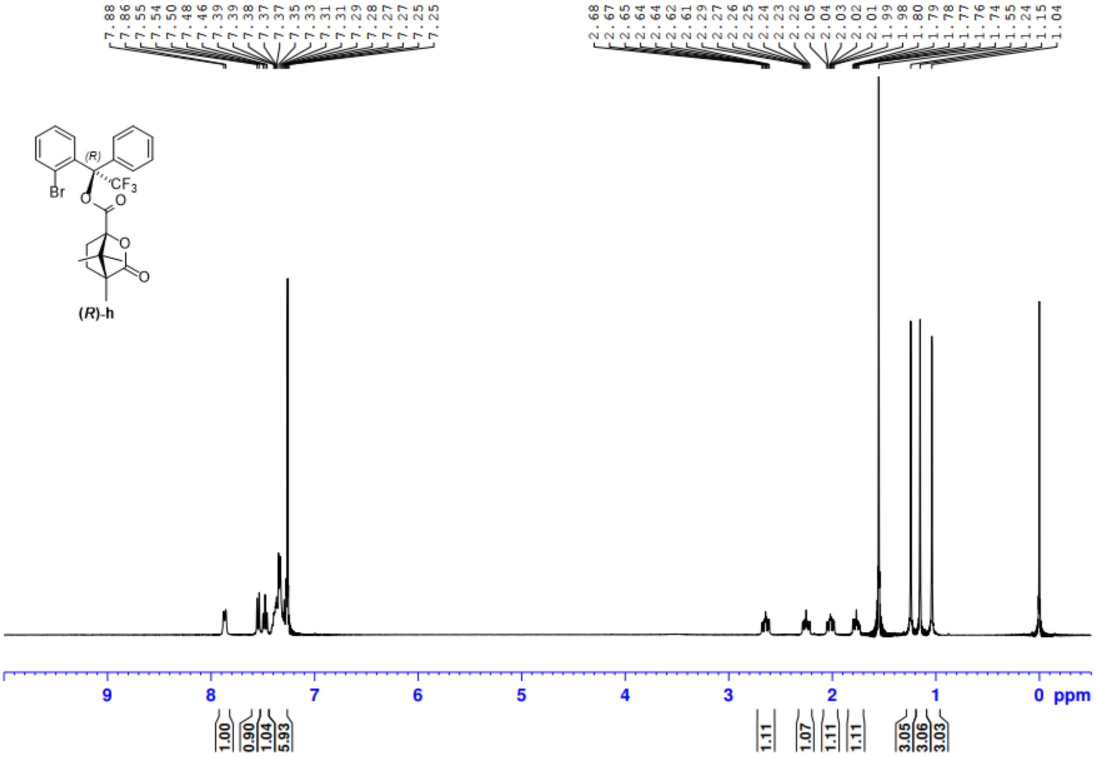

Figure S26. ^{1}H NMR of (*R*)-**h**

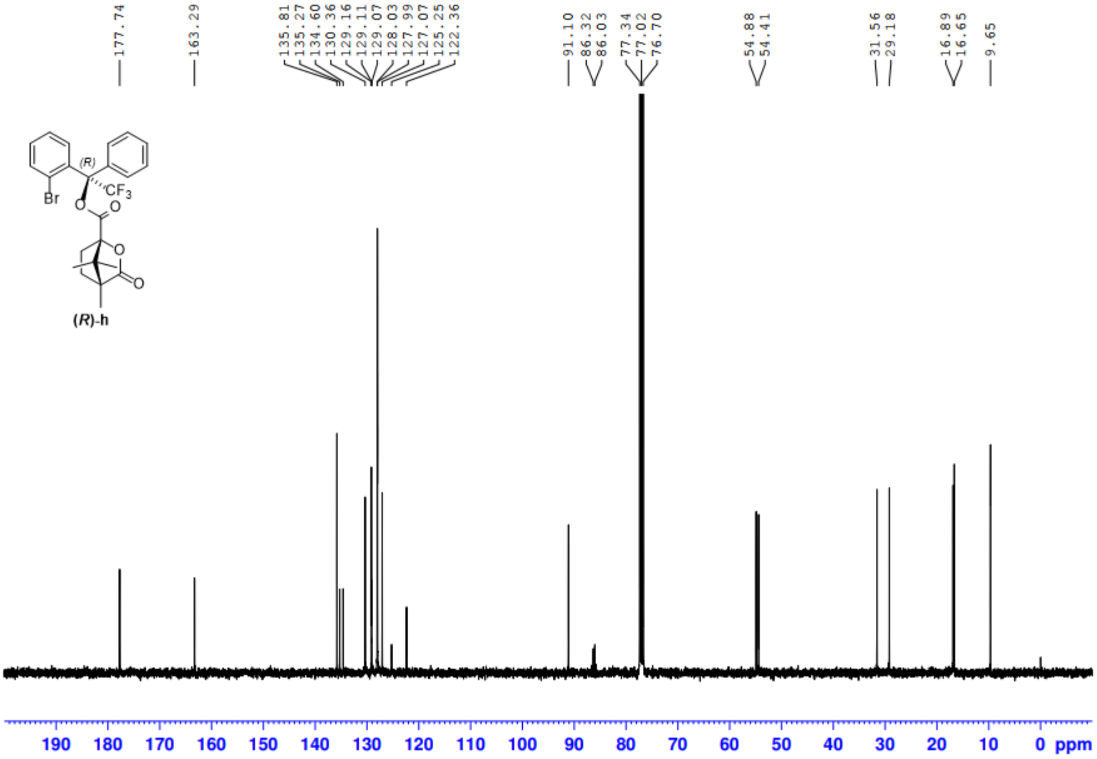

Figure S27. ^{13}C NMR of (*R*)-**h**

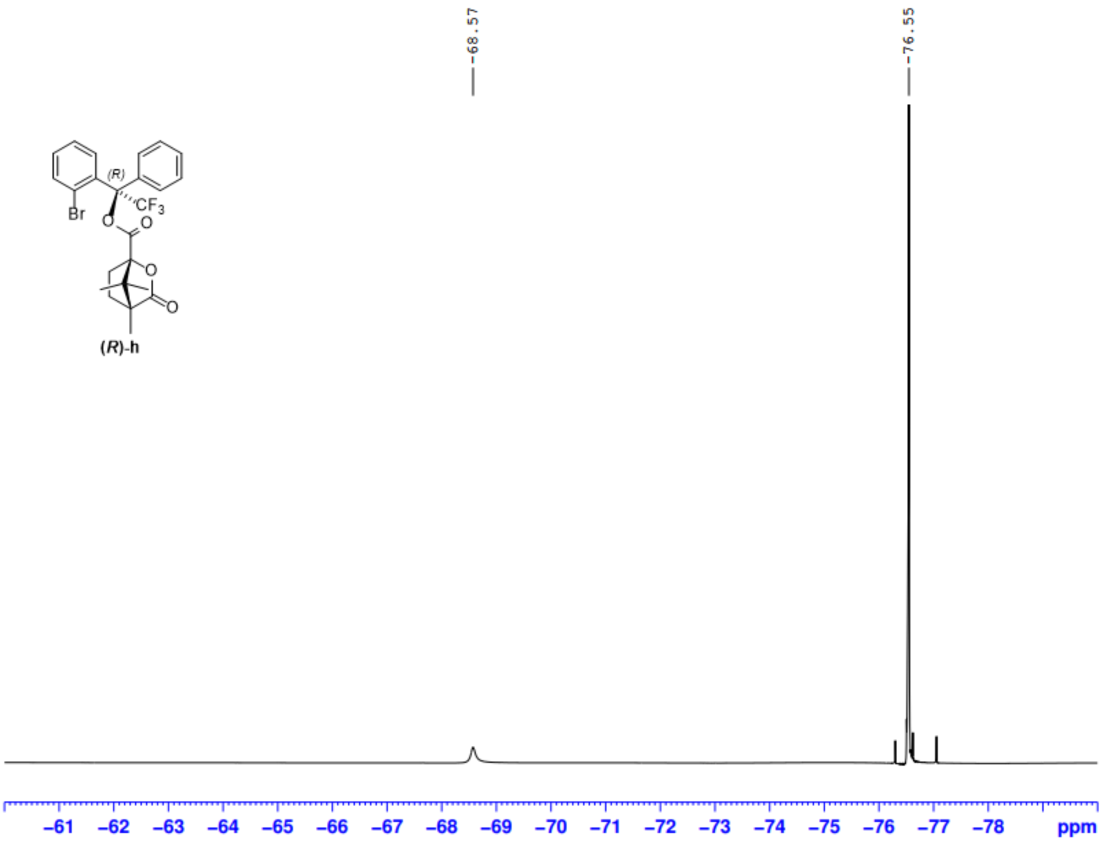

Figure S28. ^{19}F NMR of (*R*)-**h**

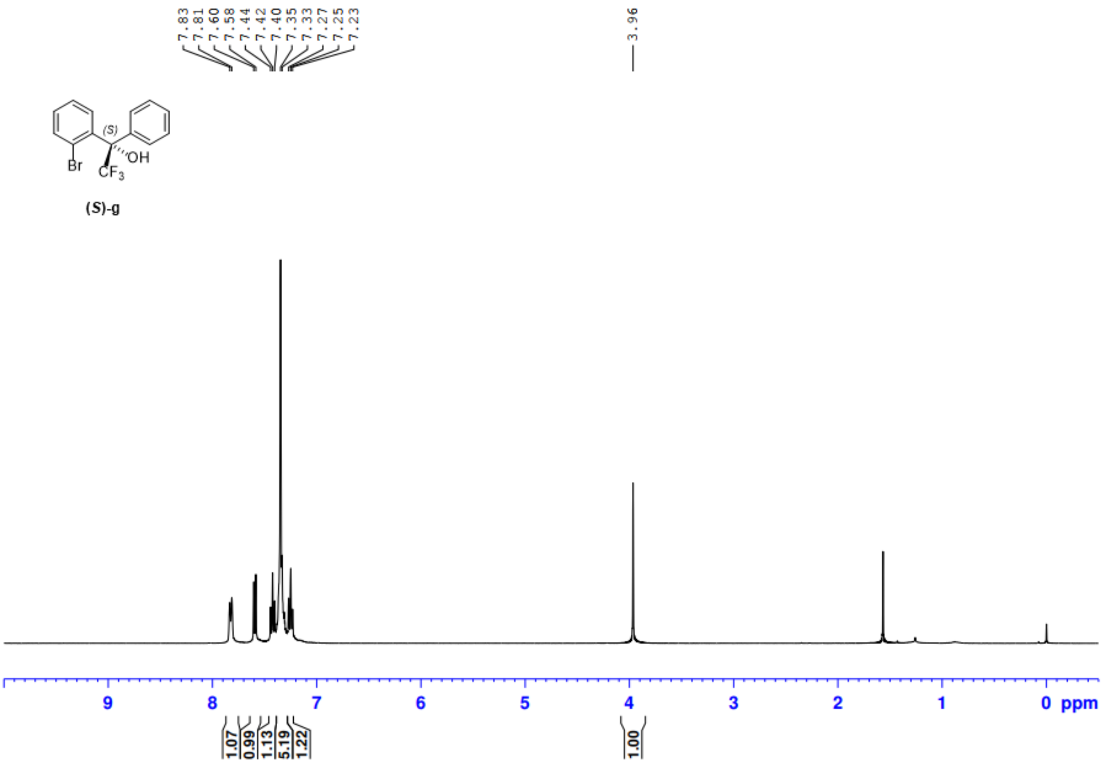

Figure S29. ^{1}H NMR of (*S*)-**g**

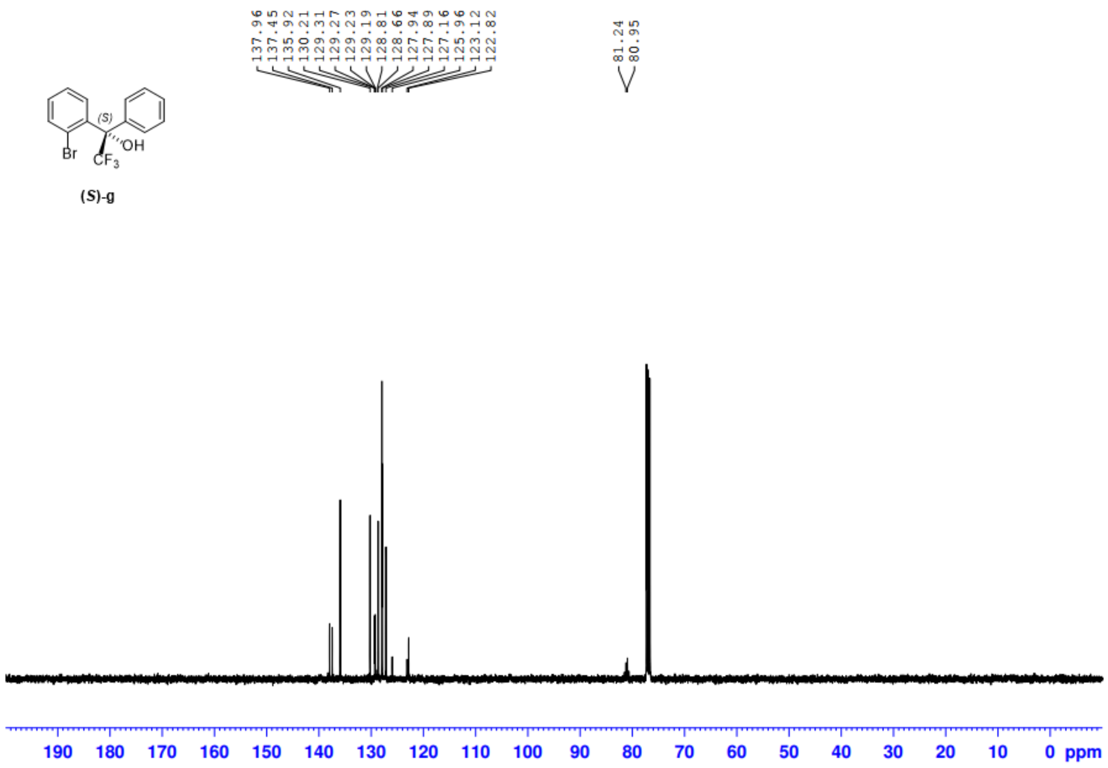

Figure S30. ^{13}C NMR of (*S*)-**g**

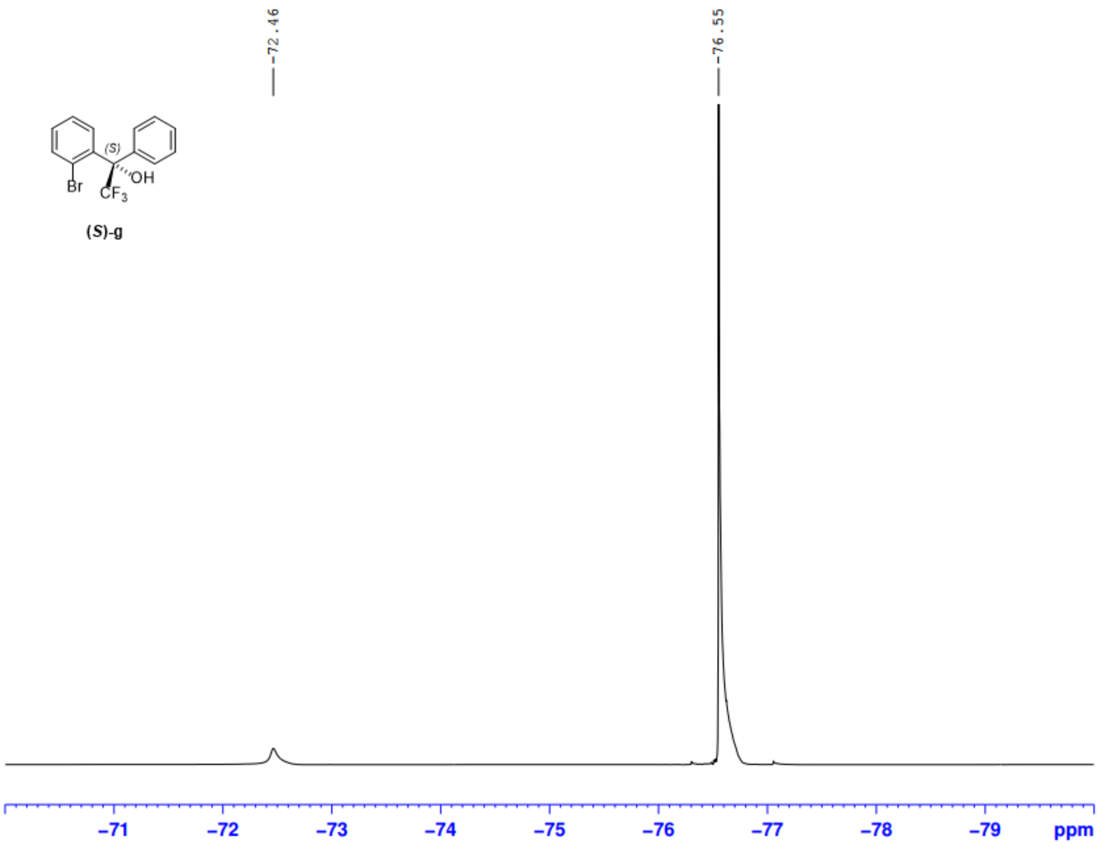

Figure S31. ^{19}F NMR of (*S*)-**g**

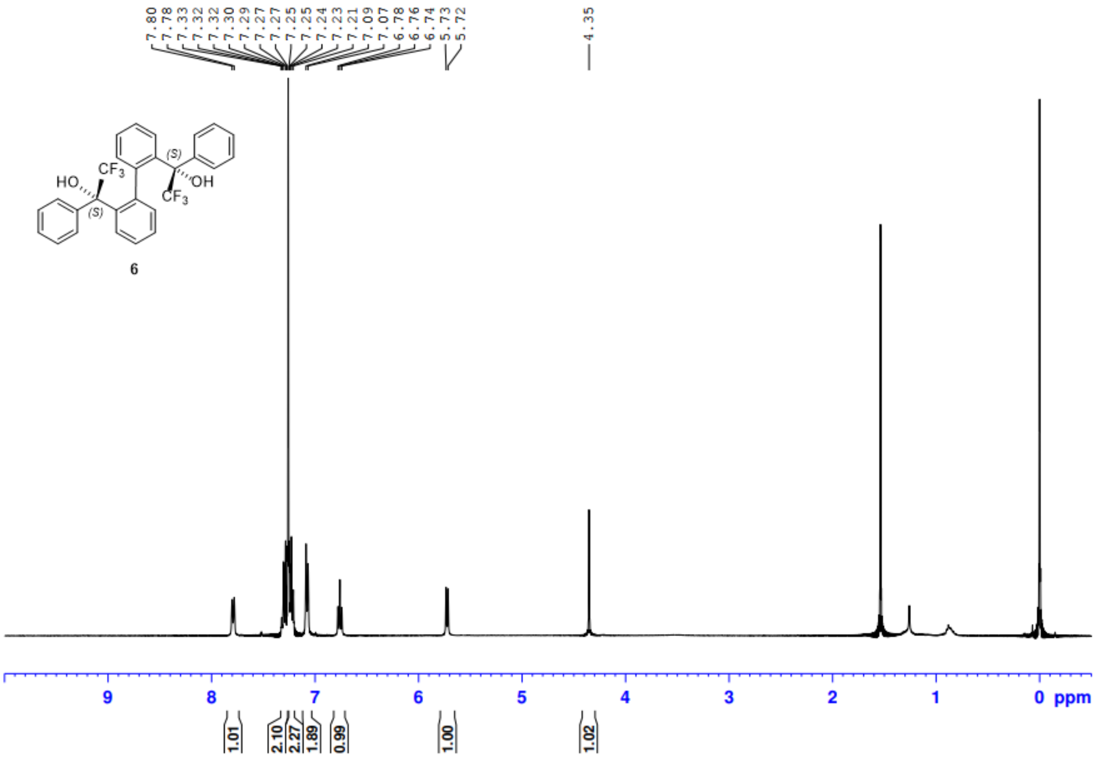

Figure S32. ^{1}H NMR of **6**

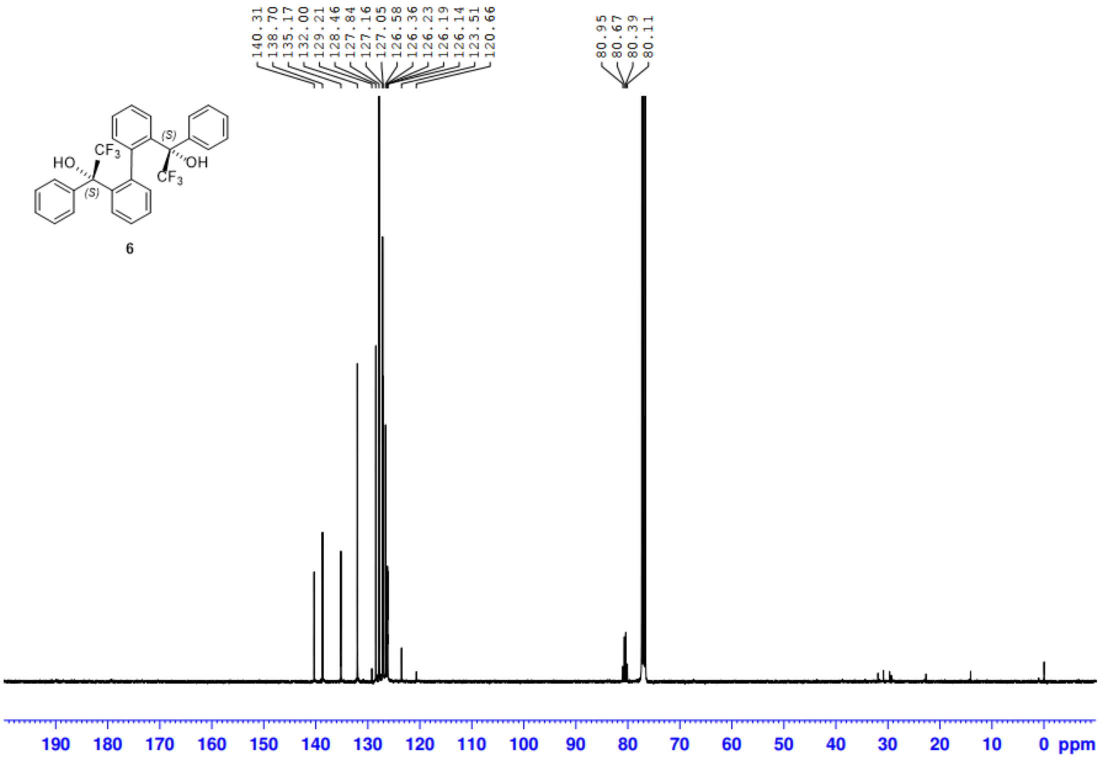

Figure S33. ^{13}C NMR of **6**

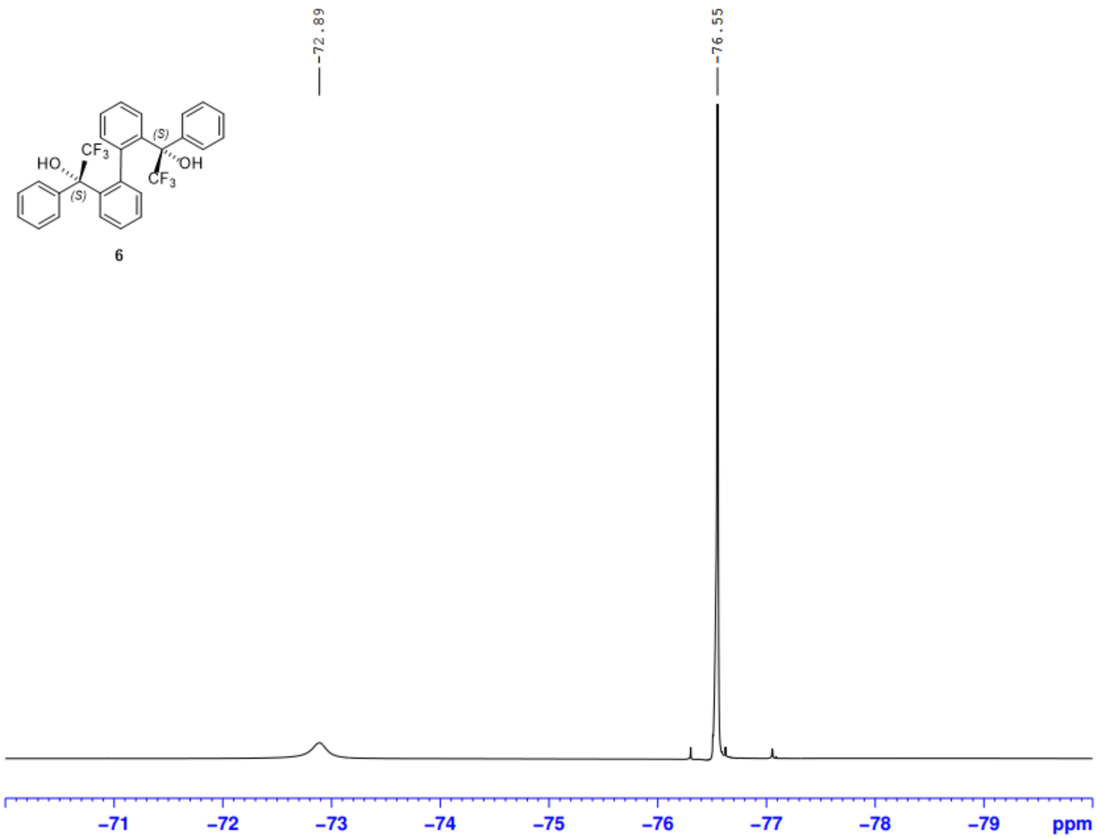

Figure S34. ^{19}F NMR of **6**

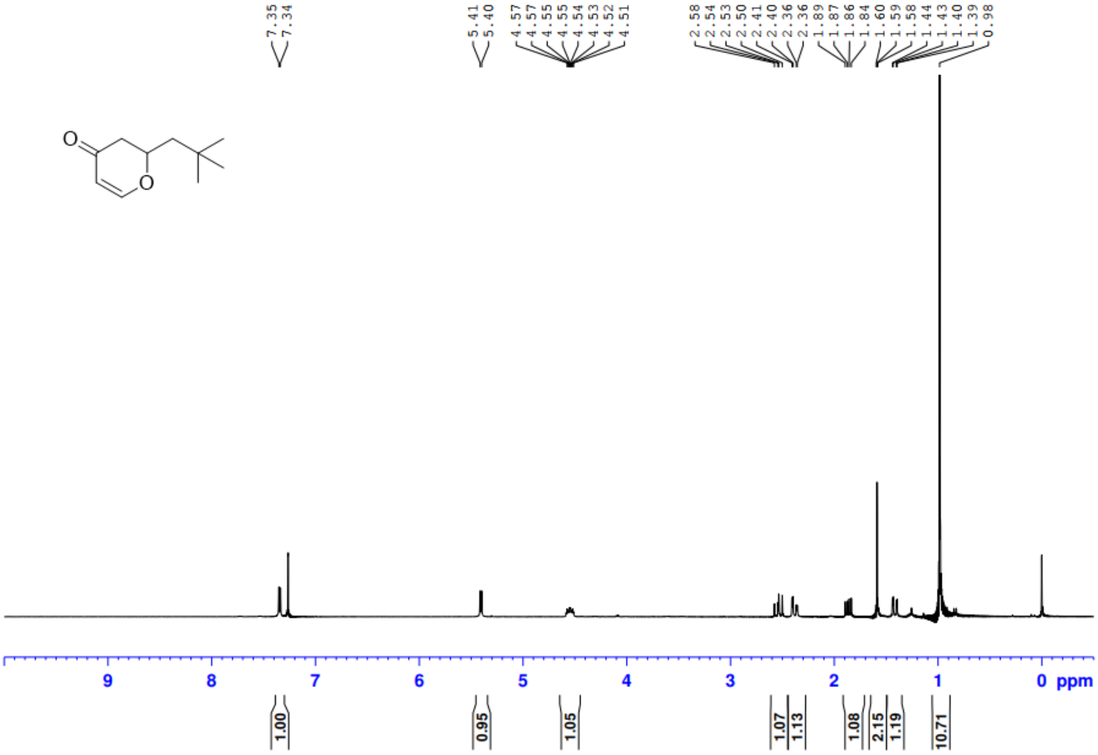

Figure S35. ^{1}H NMR of 2,3-Dihydro-2-neopentyl-4*H*-pyran-4-one

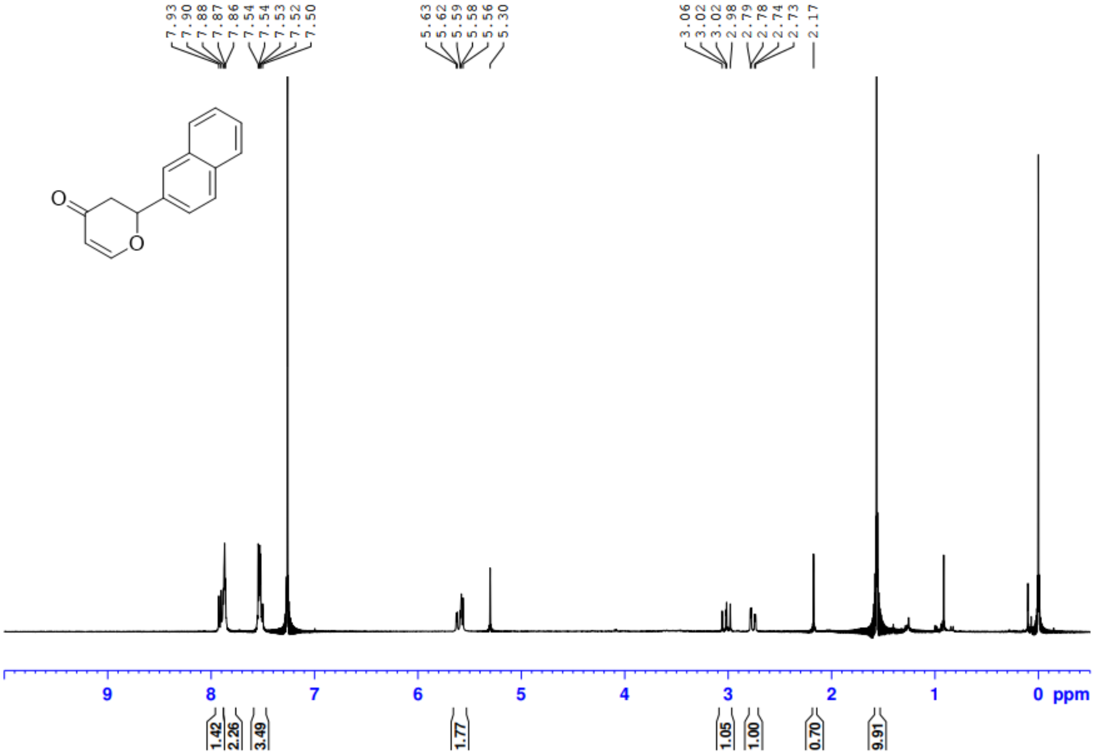

Figure S36. ^{1}H NMR 2,3-Dihydro-2-(naphthalen-2-yl)-4*H*-pyran-4-one

References:

[1] Yeung, C. T.; Yeung, H. L.; Chan, W. T. K.; Yan, S. C.; Tam, E. C. Y.; Wong, K. L.; Lee, C. S.; Wong, W. T. *CrystEngComm* **2013,** *15*, 836–840.

[2] Krause, L.; Herbst-Irmer, R.; Sheldrick, G.M.; Stalke, D. *J. Appl. Crystallogr.* **2015**, *48*, 3–10.

[3] Sheldrick, G.M. *Acta Crystallogr C* **2015**, *71*, 3–8.

[4] Unni, A. K.; Takenaka, N.; Yamamoto, H.; Rawal, V. H. *J. Am. Chem. Soc.* **2005**, 127, 1336–1337.

[5] Huang, Y.; Rawal, V. H. *J. Am. Chem. Soc.* **2002**, 124, 9662–9663.

[6] Beemelmanns, C.; Husmann, R.; Whelligan, D. K. Ozcubukcu, S.; Bolm, C. *Eur. J. Org. Chem.* **2012**, 2012, 3373–3376.

[7] Huang, Y.; Rawal, V. H. Org. Lett. **2000**, 2, 3321–3323.
